# Harnessing Botanical Extracts for Asthma Therapy: A Scoping Review of Molecular Mechanisms and the Strategic Utility of Experimental Models (2005–2025)

**DOI:** 10.3390/nu18101604

**Published:** 2026-05-18

**Authors:** Jae-Won Lee, Chang Hyeon Jeon, Soo-Jin Park, Hee Jae Lee, Hyung Won Ryu, Su Ui Lee

**Affiliations:** 1 Natural Product Research Center, Korea Research Institute of Bioscience and Biotechnology, Cheongju 28116, Republic of Korea; suc369@kribb.re.kr (J.-W.L.); wjsckdgus119@kribb.re.kr (C.H.J.); soojin921@kribb.re.kr (S.-J.P.); 2 Department of Biotechnology, University of Science and Technology, Daejeon 34113, Republic of Korea; 3 Department of Pharmacology, College of Medicine, Kangwon National University, Chuncheon 24341, Republic of Korea; heejaelee@kangwon.ac.kr; 4 Immunotherapy Research Center, Korea Research Institute of Bioscience and Biotechnology, Daejeon 34141, Republic of Korea

**Keywords:** asthma, plant extracts, airway inflammation, molecular mechanisms, bioactive compounds, translational utility

## Abstract

Asthma represents a chronic inflammatory condition of the respiratory tract, where long-term bronchial inflammation serves as a primary driver of progressive airway remodeling. This complex pathology emerges from the intricate synergy between host genetic susceptibility and diverse environmental triggers, ultimately impairing pulmonary function. At the cellular level, asthmatic responses are orchestrated by a dynamic crosstalk among various immune and structural populations, including airway epithelial cells, T-lymphocytes, eosinophils, and mast cells, which collectively perpetuate the inflammatory milieu. Although inhaled corticosteroids are the conventional cornerstone of therapy, their clinical application is frequently hindered by potential systemic toxicity and the emergence of steroid-resistant phenotypes. Consequently, botanical extracts derived from both aerial and underground plant organs have gained attention as versatile multi-target candidates capable of modulating the multifaceted pathophysiological networks of asthma. This scoping review critically synthesizes the pharmacological efficacy of these plant-based interventions in regulating pivotal signaling cascades, such as MAPK, NF-κB, STAT3/6, and GATA3. Based on a systematic literature search covering the period from 2005 to 2025, this study provides a focused quantitative analysis of preclinical literature from the last decade (2016–2025) to evaluate the in vitro and in vivo models employed to validate these therapeutic effects. The assessment reveals that the vast majority of current research continues to rely on crude botanical preparations, with only a limited subset of studies utilizing enriched fractions or fully characterized isolated compounds. This predominance of unrefined extracts underscores a significant gap in chemical standardization and highlights the necessity for more rigorous mechanistic validation. Ultimately, this paper outlines strategic pathways for translating preclinical findings into clinical practice, offering a robust framework for the development of standardized plant-derived interventions in asthma management.

## 1. Introduction

Asthma remains a pervasive global health challenge, defined by persistent endobronchial inflammation and bronchial hyperreactivity [1]. The clinical burden is exacerbated by recurrent symptomatic flares—notably dyspnea, wheezing, and paroxysmal coughing—which significantly impair health-related quality of life (HRQoL). In its most acute forms, these flares pose a substantial risk of fatality [2]. Asthmatic pathogenesis is characterized by a complex immunological cascade involving the recruitment and activation of a diverse cellular network, including airway epithelial cells (AECs) and various effector immune cells. The molecular crosstalk between these effector cells and the respiratory epithelium facilitates the liberation of diverse pro-inflammatory secretomes, which collectively instigate bronchial hyperreactivity and aberrant tissue remodeling, culminating in persistent endobronchial inflammation [3]. Among the various clinical phenotypes, allergic asthma represents the most prevalent form, characterized by a predominantly type 2 helper T cell (Th2) high-immune response to environmental allergens [4].

AECs function as the primary sentinel in asthma pathogenesis, orchestrating early immune cascades by detecting allergens and environmental triggers through pattern recognition receptors (PRRs) and Toll-like receptors (TLRs) [3]. Upon activation, these cells trigger the NF-κB and AP-1 signaling pathways to release critical alarmins—specifically IL-25, IL-33, and TSLP—which subsequently recruit effector cells such as eosinophils and mast cells to sustain a chronic Th2 inflammatory milieu. Beyond mere inflammation, AECs act as central architects of airway remodeling by driving goblet cell hyperplasia and the epithelial–mesenchymal transition (EMT) [5]. This process is further exacerbated by macrophages and eosinophils, which secrete TGF-β to stimulate fibroblastic collagen deposition, while airway smooth muscle cells (ASMCs) undergo pathological hypertrophy and proliferation [6]. Ultimately, this self-perpetuating cycle of cytopathological alterations results in impaired airway plasticity and permanent stenosis, leading to a progressive and irreversible decline in pulmonary function.

Th2 cell hyperactivation represents a cornerstone of allergic asthma, orchestrating a complex cascade through both IL-4 and IL-13 secretion, which drive immunoglobulin E (IgE) isotype switching in B cells [7]. More specifically, IL-13 acts as a potent stimulus for goblet cell hyperplasia, leading to excessive mucus production and impaired mucociliary clearance, while IL-5 and IL-9 facilitate the recruitment and activation of eosinophils and mast cells, respectively [4]. Simultaneously, the Th2-derived cytokines IL-4 and IL-13 govern the phenotypic polarization of macrophages toward a pro-fibrotic M2 state [8]. These polarized M2 macrophages function as pivotal catalysts for airway remodeling by releasing excessive TGF-β, which promotes subepithelial collagen deposition and smooth muscle hypertrophy [9]. Furthermore, the restructured airway microenvironment perpetuates chronic inflammation by secreting cytokines that re-stimulate T cell populations, establishing a self-sustaining vicious cycle [10]. While Th2-driven pathways are predominant, emerging evidence highlights the role of diverse T cell endotypes, such as Th17 cells, which induce neutrophil-dominant inflammation and corticosteroid resistance, and Th1 cells, which exacerbate airway distress during viral infections [11]. Fundamentally, failure to resolve these multifaceted inflammatory responses often stems from the functional impairment of regulatory T cells (Tregs) [12]. This breakdown in immune homeostasis not only aggravates airway hyperresponsiveness (AHR) but also ensures that T cell-derived mediators continuously drive the structural alterations and epithelial damage that define the transition to chronic, irreversible asthma.

Within the asthmatic inflammatory milieu, B lymphocytes (B cells) function as indispensable effectors by orchestrating antibody production and amplifying immune signaling. Driven by Th2-derived cytokines such as IL-4 and IL-13, B cells undergo isotype switching to synthesize allergen-specific IgE, a pathognomonic hallmark of atopic asthma [7]. This secreted IgE sensitizes mast cells by binding to high-affinity receptors (FcεRI); upon subsequent allergen re-exposure, this complex triggers the degranulation of potent mediators like histamine, initiating immediate hypersensitivity [13]. Beyond their humoral role, B cells act as professional antigen-presenting cells (APCs) that capture and process exogenous antigens for T cell recognition, thereby sustaining a reciprocal activation loop that perpetuates chronic airway distress [14]. Furthermore, activated B cell subsets contribute to the inflammatory microenvironment by secreting diverse cytokines that recruit additional leukocytes to the pulmonary interstitium [15]. Consequently, B cells bridge the gap between acute allergic triggers and persistent inflammation, making their IgE-dependent pathways a primary target for biologic interventions, such as omalizumab, in refractory asthma management.

Eosinophils function as primary effector cells in the asthmatic airway, driving tissue damage through the targeted release of potent cytotoxic granules. Upon degranulation, preformed cationic proteins—such as major basic protein (MBP) and eosinophil cationic protein (ECP)—directly disrupt the airway epithelium’s integrity, thereby fostering AHR [16]. Beyond acute cytotoxicity, eosinophils propagate persistent inflammation and edema by synthesizing lipid mediators, notably cysteinyl leukotrienes (CysLTs), which exert potent bronchoconstrictor effects and enhance vascular permeability [17]. These biochemical shifts result in mucosal swelling and hypersecretion, further narrowing the airway lumen. Moreover, in accelerating airway remodeling, eosinophils are critical contributors to asthma chronicity. By secreting key growth factors, particularly TGF-beta, they stimulate bronchial wall thickening and rigidification through subepithelial fibrosis [18]. Consequently, eosinophils transition from mediators of acute exacerbation to drivers of permanent physiological decline, orchestrating the irreversible structural changes that define severe asthma.

Mast cells function as primary initiators of the immediate asthmatic response, driving rapid bronchial constriction upon allergen exposure. This process is triggered when allergen-specific IgE cross-links with high-affinity receptors on the mast cell surface, inducing the explosive release of preformed mediators such as histamine and newly synthesized leukotrienes [13]. These agents exert potent contractile effects on bronchial smooth muscle, manifesting as acute wheezing and dyspnea within minutes. Beyond bronchoconstriction, mast cells discharge lipid mediators, such as prostaglandin D2 (PGD2), which facilitate vasodilation and mucosal edema [19]. Such alterations not only exacerbate airway narrowing but also prime the pulmonary microenvironment for the influx of additional inflammatory effectors [20]. Crucially, mast cells serve as a functional bridge between acute hypersensitivity and chronic inflammation by secreting a diverse array of cytokines, including TNF-α, IL-4, and IL-5 [21]. This signaling cascade perpetuates disease by continuously stimulating T cells and eosinophils, ensuring the progression of asthma beyond the initial phase [22]. Notably, the mast cell-induced release of TNF-α and IL-5 acts as a powerful chemotactic signal, actively recruiting eosinophils from the systemic circulation into the airway interstitium [23]. Upon arrival, these recruited eosinophils unleash cytotoxic proteins, resulting in extensive epithelial devastation and structural compromise [24]. Eventually, mast cells are indispensable regulators that orchestrate both the immediate allergic flare and long-term inflammatory persistence in asthma.

Although macrophages are fundamentally tasked with maintaining immunological homeostasis and airway clearance, they emerge as central orchestrators of asthma pathophysiology by perpetuating chronic inflammatory cascades. Upon adopting an M1 phenotype, these cells detect exogenous pathogens or irritants and secrete key pro-inflammatory cytokines—including TNF-α, IL-1β, and IL-6—thereby initiating and amplifying the early-stage inflammatory response through recruiting diverse leukocyte populations [25]. Conversely, the M2 phenotype, which predominates in the asthmatic pulmonary environment, drives the transition toward chronicity and structural modification [26]. By releasing TGF-β, M2-polarized macrophages facilitate subepithelial fibrosis, culminating in the progressive thickening and stiffening of the bronchial walls—a pathological hallmark known as airway remodeling that leads to a permanent decline in respiratory function [27]. Crucially, asthmatic macrophages exhibit a profound impairment in efferocytosis and phagocytic capacity, hindering the effective removal of apoptotic cells and antigenic debris [28]. Within the respiratory tract, the persistence of these un-cleared waste products provides a continuous inflammatory stimulus, which contributes to intractable complications and symptom persistence [29]. The aberrant activation and functional exhaustion of macrophages thus play a decisive role in both propagating persistent inflammation and catalyzing the irreversible airway restructuring.

While synthetic medications are fundamental to current asthma management, their long-term application can be hindered by various side effects and reduced efficacy in certain patient groups, such as those with steroid-resistant symptoms or fatality [30]. Common concerns, ranging from local throat irritation to broader systemic issues, highlight the importance of exploring new therapeutic avenues [31]. In this context, natural plant extracts are gaining attention as promising complementary options, offering diverse bioactive compounds that may address the limitations of conventional treatments and support more comprehensive respiratory care and vitality [32].

The diverse array of secondary metabolites sequestered within botanical leaves, stems, roots, rhizomes, flowers, fruits, and bark exhibits a broad spectrum of biological activities, including antioxidant, anti-inflammatory, immunomodulatory, and epithelial-protective effects [33]. However, interpretation of botanical asthma studies remains incomplete when biological outcomes are reported only at the crude-extract level. To clarify the mechanistic relevance of these extracts, it is necessary to identify the major phytochemical markers, classify them into secondary-metabolite groups, and relate these constituents to recurrent asthma-relevant targets such as NF-κB, MAPK, STAT3/6, GATA3, MUC5AC, TSLP, IgE, and Th2 cytokines. Accordingly, this review provides a focused synthesis of botanical extract studies published over the past decade, with particular attention to plant-part origin, extraction solvent, extraction yield when reported, extract or fraction type, solvent polarity, phytochemical characterization, asthma-relevant in vitro and in vivo models, inflammatory stimuli, and pathway-level mechanisms. This analytical framework is intended to clarify not only whether botanical preparations exhibit anti-asthmatic activity, but also how extraction-related variables and chemical characterization influence reproducibility, biological interpretation, and translational prioritization. This analytical scope is intended to clarify not only whether botanical extracts exhibit anti-asthmatic activity but also which experimental systems and molecular endpoints most consistently support their translational prioritization. Furthermore, this review provides a structured overview of the preclinical in vitro and in vivo frameworks employed to validate these botanical interventions. By offering these methodological blueprints, this paper aims to assist asthma researchers in constructing more refined and robust experimental designs.

Several reviews have already addressed the anti-asthmatic potential of natural products and phytochemicals. Park et al. summarized the roles of phytochemicals in bronchial asthma, with emphasis on anti-oxidative, anti-inflammatory, and anti-vascular actions [34]. Amaral-Machado et al. provided a broader overview of natural products in asthma treatment, including plant-, animal-, and microorganism-derived products [35]. More recently, Jasemi et al. reviewed phytochemicals for allergic asthma and their therapeutic mechanisms [36], whereas Rajizadeh et al. comprehensively updated plants and herbal compounds with anti-asthmatic effects [37].

These publications collectively establish the therapeutic relevance of natural products in asthma; however, they do not specifically organize recent evidence according to botanical plant part, extract/fraction type, preclinical model, inflammatory stimulus, and molecular endpoint. Therefore, the novelty of the present review does not lie in introducing plant-derived products as a new topic, but in providing a plant-part-resolved and model-oriented analysis of botanical extracts evaluated during the last decade. Specifically, this review focuses on extracts from aerial and underground plant organs and maps their effects onto asthma-relevant readouts, including Th2 cytokines, IgE, eosinophilic infiltration, mucus production, airway hyperresponsiveness, remodeling markers, and signaling axes such as NF-κB, MAPK, STAT3/6, GATA3, and TSLP. By linking botanical source, experimental platform, and mechanistic endpoint, this review fills a conceptual and methodological gap between broad phytochemical catalogues and practical preclinical study design for asthma therapy. Ultimately, this synthesis underscores the translational potential of plant-derived extracts as either supportive or innovative paradigms in the clinical management of asthma.

## 2. Methodology

To ensure the scientific rigor and reproducibility of this review, we conducted a structured search and selection of the literature as follows:

Review Type: This study is defined as a scoping review, not a systematic review or meta-analysis. It incorporates elements of structured search and quantitative synthesis to map the existing evidence on the molecular signaling pathways and translational value of botanical extracts in asthma.

Database: A comprehensive search was performed exclusively using PubMed/MEDLINE, the primary database for biomedical and pharmacological research.

Time Window: A two-track chronological approach was employed to ensure both contextual depth and analytical precision. The overall search encompassed articles published between January 2005 and December 2025 to provide a broad thematic background. Within this framework, a focused quantitative analysis was conducted on preclinical literature from the last decade (2016–2025) to reflect the most recent mechanistic advancements and experimental trends. References from 2005–2015 were used mainly for background/context, whereas 2016–2025 preclinical studies were used for the core quantitative mapping.

Search Terms and Boolean Operators: We used a combination of MeSH terms and relevant keywords with Boolean operators: ((“Botanical extract” OR “Plant extract” OR “Herbal medicine”) AND (“Asthma” OR “Airway inflammation”)) AND (“Molecular mechanism” OR “Signal transduction” OR “Translational model”).

Inclusion Criteria: (1) Peer-reviewed original research and reviews; (2) Studies focusing on botanical extracts or their active compounds; (3) Research utilizing in vitro or in vivo (e.g., OVA-induced) asthma models; (4) Articles published in English.

Exclusion Criteria: (1) Conference abstracts, editorials, and posters; (2) Studies without a clear focus on the molecular mechanism of asthma; (3) Clinical trials without mechanistic data.

Study Selection Procedure: Initially, titles and abstracts were screened for relevance. Subsequently, 120 full-text articles (including foundational guidelines and the most recent 2025 studies) were selected to evaluate their contribution to the mechanistic insights and translational value presented in this manuscript. The terminology and methodological criteria applied in this selection process strictly fulfill the requirements for a scoping review.

## 3. Molecular Orchestration of Airway Inflammation: Regulatory Crosstalk Between NF-κB, MAPK, and STAT Signaling Pathways

The pathogenesis of asthma is fundamentally rooted in the aberrant activation of Th2-mediated immune responses, where the NF-κB, MAPK, and STAT3/6 signaling pathways function as pivotal molecular checkpoints [38,39,40]. By intercepting these interconnected cascades, botanical-derived bioactive compounds offer a multi-targeted therapeutic approach. More specifically, these extracts inhibit the nuclear translocation of NF-κB and the phosphorylation of STAT6, both of which are essential for the transcriptional upregulation of GATA3, the master regulator of Th2 cell commitment. This dual inhibition effectively suppresses the polarization of naive T cells, thereby curtailing the secretion of type 2 cytokines, including IL-4, IL-5, and IL-13 [41]. Furthermore, the attenuation of MAPK (ERK, JNK, and p38) and STAT3 signaling serves to dampen eosinophil and mast cell effector functions. While MAPK inhibition reduces IgE-mediated degranulation and eosinophil infiltration, STAT3 signaling modulation is crucial for mitigating epithelial-to-mesenchymal transition and subepithelial fibrosis [42,43]. By synergistically regulating these pathways, botanical interventions not only diminish mucus hypersecretion and mucosal edema in the airway epithelium but also alleviate AHR. Consequently, this integrative immunomodulation stabilizes the pulmonary microenvironment, providing a clinical basis for relieving symptomatic distress such as dyspnea and chronic cough [38,44,45,46].

Despite the widespread clinical utility of glucocorticoid receptor (GR)-targeted treatments, the increasing burden of steroid-resistant asthma underscores the critical need for alternative therapeutic strategies [30]. More specifically, there is an urgent demand for novel agents that can concurrently address both persistent inflammation and structural airway remodeling [47]. In this regard, botanical extracts—particularly those derived from leaves and roots—emerge as promising candidates, offering a multifaceted pharmacological profile that may either circumvent the limitations of traditional corticosteroids or act synergistically with them.

## 4. Rationale for Selecting Cell Lines and Stimuli in Asthma Research

To evaluate the in vitro anti-inflammatory and -asthmatic potential of the extracts and their constituent compounds, RAW 264.7 murine macrophages and NCI-H292 human airway epithelial cells were employed as representative cellular models [48,49]. RAW 264.7 cells serve as a critical model for investigating immune hypersensitivity, as macrophages are primary immune effectors that release a cascade of pro-inflammatory cytokines (e.g., TNF-α and IL-6) in response to external antigens [50]. This model allows us to assess how effectively the extracts can suppress excessive immune cell activation.

Retaining the essential characteristics of the human tracheobronchial epithelium, NCI-H292 cells serve as the primary platform for investigating MUC5AC-mediated mucus hypersecretion due to their robust response to Th2 cytokines [51,52,53]. While BEAS-2B cells are used extensively to model airway inflammation, their application in evaluating mucus production is relatively limited in asthma-related extract studies, as they often exhibit lower MUC5AC expression levels compared to NCI-H292 cells under typical asthmatic stimuli. Additionally, A549 human alveolar epithelial cells were utilized to examine inflammatory mediators and cellular adhesion within the lower respiratory environment [48]. In these cell lines, asthmatic conditions were induced using specific stimuli: LPS, TNF-α, IL-4, IL-13 and PMA. A potent endotoxin derived from bacterial cell walls, LPS, was utilized to stimulate RAW 264.7 cells, effectively recapitulating the cytokine storm and localized inflammatory environment observed in the lungs of asthmatic patients. Meanwhile, TNF-α, IL-4, IL-13 and PMA were employed to activate the NF-κB and protein kinase C (PKC) pathways, respectively. These stimuli are well-established for inducing mucus overproduction and cell adhesion molecule upregulation, i.e., ICAM-1, in NCI-H292 and A549 cells. Collectively, these models mimic the complex pathophysiology of airway obstruction, enabling a comprehensive evaluation of the extracts’ ability to mitigate both the immunological and structural hallmarks of asthma.

## 5. Connecting In Vitro Findings to the OVA-Induced Murine Model

The primary rationale for extrapolating these in vitro results to the OVA-induced murine asthma model lies in the simplification and mechanistic validation of complex systemic responses. Integrating cellular and animal models establishes several critical translational links:

Firstly, cellular assays serve as a functional bridge for validating immune cell infiltration. In the OVA-sensitized lung environment, a hallmark feature is the massive influx of macrophages and eosinophils into the pulmonary tissue. The observation that a therapeutic extract reduces pro-inflammatory cytokines in LPS-stimulated RAW 264.7 cells strongly implies its potential to decrease inflammatory mediators within the bronchoalveolar lavage fluid (BALF) of OVA-challenged mice, thereby predicting systemic anti-inflammatory efficacy [48].

Secondly, these cellular models provide a robust mechanistic basis for interpreting the complex features of airway inflammation and AHR. While asthmatic conditions in vivo are characterized by bronchial edema and heightened sensitivity, the suppression of epithelial damage and MUC5AC-mediated mucus production in PMA-, IL-4/IL-13-, or TNF-α-stimulated NCI-H292 and A549 models establishes a compelling rationale for the histological improvements observed in OVA-challenged mice. More specifically, the inhibitory effects demonstrated in these in vitro platforms directly correlate with the reduced goblet cell hyperplasia and inflammatory cell infiltration typically identified via PAS lung tissue staining [52]. These models therefore bridge the gap between molecular signaling and physiological manifestations, enabling a comprehensive evaluation of the extracts’ therapeutic efficacy.

**Figure 1:**
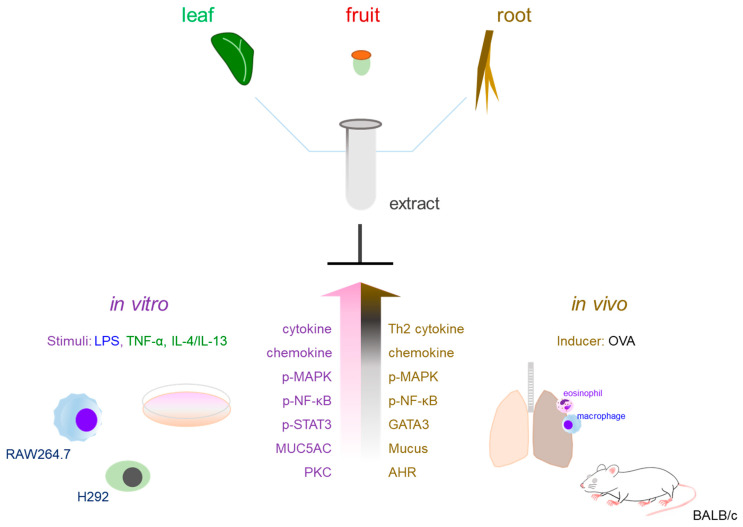
Anti-asthmatic efficacy of plant extracts and the translational utility of integrated in vitro and in vivo models. In RAW 264.7 murine macrophages, LPS triggers a potent inflammatory cascade by interacting with TLR4, a process that subsequently recruits the MyD88 adaptor protein to initiate downstream signaling. This molecular interaction triggers the simultaneous activation of the NF-κB and MAPK pathways (including ERK, JNK, and p38). The synergistic activation of these signaling axes leads to the elevated production of key inflammatory mediators such as NO and PGE2, alongside a repertoire of pro-inflammatory cytokines such as TNF-α, IL-6, and IL-1β. This model effectively recapitulates the hyper-inflammatory microenvironment observed in the airways of asthmatic patients, providing a comprehensive platform for evaluating the anti-asthmatic efficacy of therapeutic extracts. In parallel, the application of IL-4, IL-13, TNF-α and PMA to airway epithelial cell lines, such as H292, serves to simulate the allergic microenvironment in vitro. Specifically, IL-4-/IL-13- or TNFα-stimulated H292 cells effectively mimic critical features of asthma, including mucus hypersecretion (evidenced by upregulated MUC5AC expression), the release of pro-inflammatory mediators, and the disruption of intercellular junctions. These cellular models provide a robust platform for evaluating the inhibitory effects of extracts on airway remodeling and excessive pulmonary mucus production. The anti-inflammatory effects observed in these in vitro systems are closely correlated with the therapeutic outcomes in OVA-induced sensitization and challenge models. More specifically, pro-inflammatory cytokine and mucus-related marker suppression in vitro provides a mechanistic rationale for the reduced AHR and pulmonary cell infiltration observed in vivo. Ultimately, NF-κB and MAPK signaling inhibition serves as a mechanistic proxy for the Th2-driven allergic responses in OVA-induced asthma models. By targeting these key regulatory nodes, therapeutic extracts provide a solid foundation for alleviating structural airway remodeling and pulmonary obstruction in living organisms.

Finally, cellular signaling serves as a surrogate indicator for the prototypical Th2-mediated immune cascade. Since the OVA model represents a complex Th2-driven allergic response, the ability of an extract to block specific NF-κB or MAPK pathways in in vitro models (induced by LPS, PMA, or Th2 cytokines such as IL-4/IL-13) indicates its capacity to intercept key nodes within the multifaceted immune network. Consequently, these in vitro platforms do not merely streamline the initial screening process; they provide mechanistic evidence essential for understanding how therapeutic interventions mitigate allergic airway remodeling and pulmonary obstruction in vivo. To provide a structured overview of these paradigms, the experimental methodologies, molecular targets, and their respective therapeutic implications are quantitatively synthesized in Table 1 and Table 2, with further mechanistic details illustrated in Figure 1.

## 6. Modulatory Effects of Extracts from Different Plant Parts on Airway Inflammation and Cytokine Expression in Asthma

This section provides a comprehensive analysis of 39 studies evaluating botanical preparations and plant-derived constituents for anti-asthmatic efficacy across diverse experimental frameworks. Because the degree of chemical definition varied substantially among studies, the tested materials were stratified into five categories: crude extracts without reported phytochemical markers, crude extracts with phytochemical or chromatographic characterization, extract-plus-isolated-compound studies, enriched fractions, and isolated compounds. This classification allows the evidence base to be interpreted not only descriptively but also in terms of the chemical definition and reproducibility of the tested materials. More specifically, the pharmacological profiles of approximately 40 extracts derived from various plant parts—including leaves, roots, and fruits—are detailed, with an emphasis on their modulatory effects on key inflammatory markers and signaling pathways in both in vitro and in vivo models. For a more intuitive grasp of these findings, detailed summaries regarding specific plant parts, their therapeutic mechanisms, and the corresponding in vitro and in vivo models—along with their associated key parameters—are provided in Table 3, Table 4, Table 5, Table 6, Table 7 and Table 8. In this section, botanical extracts are organized into Section 6.1, Section 6.2, Section 6.3, Section 6.4, Section 6.5, Section 6.6, Section 6.7, Section 6.8, Section 6.9, Section 6.10, Section 6.11, Section 6.12, Section 6.13, Section 6.14, Section 6.15, Section 6.16, Section 6.17, Section 6.18, Section 6.19, Section 6.20, Section 6.21, Section 6.22, Section 6.23, Section 6.24, Section 6.25, Section 6.26, Section 6.27, Section 6.28, Section 6.29, Section 6.30, Section 6.31, Section 6.32, Section 6.33, Section 6.34, Section 6.35, Section 6.36, Section 6.37, Section 6.38, Section 6.39 and Section 6.40.

### 6.1. Phytochemical Context, Extraction Characteristics, and Quantitative Stratification of Tested Materials

Across the studies reviewed in this section, the anti-asthmatic effects of botanical preparations were most frequently associated with phenolic acids, flavonoids and flavonoid glycosides, coumarins, iridoid glycosides, saponins, alkaloids, limonoids, catechins, phenylethanoid glycosides, diarylheptanoids, and sesquiterpene lactones. Phenolic acids and flavonoids, including chlorogenic acid, caffeic acid, p-coumaric acid, isoquercetin, hyperoside, quercetin and kaempferol rhamnosides, naringenin, catechin, and catechin derivatives, were repeatedly linked to the suppression of NF-κB/MAPK activation, oxidative stress, inflammatory cytokine release, and mucus-related endpoints.

In contrast, alkaloid- and limonoid-rich preparations, such as Fritillaria total alkaloids, Alstonia total alkaloids, Dictamnus limonoids/alkaloids, and Phellodendron alkaloids, were more commonly associated with broader immunomodulatory effects involving Th2/Th17 cytokines, IgE, TSLP, TRPV1/NFAT, STAT3/6, and leukotriene-related pathways. Iridoid glycosides, saponins, and sesquiterpene lactones were also recurrently implicated in the regulation of Th2 cytokine responses, epithelial mucus production, and JAK/STAT signaling.

However, the strength of phytochemical evidence varied considerably across studies. In several cases, isolated or purified constituents were directly evaluated, including osthole, dehydromatricarin A, sophoricoside, gypenoside A, Fritillaria total alkaloids, Alstonia total alkaloids, and constituents isolated from Inula japonica. In other studies, phytochemicals were identified only as chromatographic markers of active extracts, and their causal contribution to the observed anti-asthmatic effects remains putative. Therefore, throughout this review, identified metabolites are interpreted as candidate contributors or standardization markers unless direct pharmacological testing of the isolated compound was performed.

Before discussing individual botanical sources, the reviewed studies were categorized according to the nature of the tested materials and the degree of chemical definition. For this purpose, “crude extracts” were defined as solvent, aqueous, or hydroalcoholic preparations evaluated without purification into a defined chemical fraction. “Chemically characterized crude extracts” were defined as crude extracts accompanied by phytochemical marker identification, chromatographic profiling, or quantitative phytochemical analysis. “Standardized extracts” were defined more strictly as preparations for which quantitative marker specifications, batch-to-batch reproducibility, or defined standardization criteria were reported. “Enriched fractions” referred to partially purified preparations enriched in a specific chemical class, such as total alkaloid fractions. “Isolated compounds” referred to purified single constituents evaluated as pharmacological interventions.

Extraction-related information was additionally extracted from the original studies whenever available. The following variables were recorded: plant part used, extraction solvent, extraction yield, extraction or fractionation method, degree of phytochemical characterization, and whether the preparation corresponded to a hydrophilic, hydroalcoholic/intermediate-polarity, lipophilic, crude, or partially purified material. In this review, water extracts were classified as hydrophilic preparations; ethanol, methanol, and hydroalcoholic extracts were considered intermediate-polarity or broadly polar organic extracts; and ethyl acetate, chloroform, *n*-butanol, petroleum ether, hexane, essential oil, or oil-based preparations were classified according to their relative polarity and fractionation status. Total alkaloid fractions and other class-enriched preparations were classified as partially purified or enriched fractions rather than crude extracts. When the extraction yield, solvent ratio, extraction temperature, extraction time, or solid-to-liquid ratio was not available in the original article, this was recorded as “not reported.”

Among the 39 studies summarized in Section 5, 15 used crude extracts without reporting specific phytochemical markers or chromatographic characterization, whereas 17 used crude extracts accompanied by some degree of phytochemical or chromatographic characterization. From an extraction-reporting perspective, plant part information was available for most studies, but extraction yield and detailed extraction conditions were inconsistently reported. In particular, many studies described the tested material only as an ethanol extract, methanol extract, aqueous extract, or total alkaloid fraction without providing extraction yield, solvent-to-material ratio, extraction duration, temperature, or batch reproducibility data. This limitation is important because the extraction solvent and solvent polarity strongly influence the relative enrichment of hydrophilic phenolics, glycosides, saponins, alkaloids, lipophilic terpenoids, limonoids, and other secondary metabolites. Therefore, extraction variables were treated as interpretive factors throughout the review and were summarized separately in the revised tables. However, based on the information available in the reviewed studies, few, if any, extracts could be considered fully standardized according to quantitative marker specifications and batch reproducibility criteria. In addition, 2 studies evaluated both crude extracts and isolated constituents, 2 studies tested enriched total alkaloid fractions, and 3 studies examined isolated compounds alone. Overall, crude extract-based investigations accounted for 32 of the 39 studies, representing 82.1% of the reviewed literature. Regarding chemical characterization, 21 of the 39 studies, or 53.8%, reported phytochemical markers or chromatographic characterization of an extract or fraction, whereas 15 studies, or 38.5%, lacked explicit chemical characterization. When isolated compounds are also considered chemically defined materials, 24 of the 39 studies, or 61.5%, provided at least some degree of chemical definition. These findings indicate that the current literature remains heavily dependent on crude or partially characterized botanical preparations and that rigorous phytochemical standardization remains a major limitation in preclinical asthma research.

### 6.2. Pistacia integerrima

Traditionally, *P. integerrima* has been utilized to manage respiratory conditions such as cough and asthma [90]. A prior study by Rana et al. investigated the therapeutic potential of *P. integerrima* ethanol extract (EE) [PIEE] using an ovalbumin (OVA)-sensitized murine model of asthma [75]. Their findings demonstrated that intranasal treatment with 200 mg/kg of PIEE effectively mitigated lung goblet cell hyperplasia and the infiltration of inflammatory cells—including eosinophils, neutrophils, monocytes, and lymphocytes—in both blood and BALF. Furthermore, PIEE treatment normalized the expression of inflammatory cytokines (TNF-α, IL-4, and IL-5) and water channel proteins (AQP-1 and AQP5) at the mRNA level, while also reducing pulmonary edema, as evidenced by a lower lung wet/dry weight ratio. These effects were equivalent to those observed with 15 mg/kg of methylprednisolone (MP), a standard reference drug frequently employed to treat allergic airway inflammation.

### 6.3. Erythronium japonicum

A medicinal herb traditionally utilized across East Asia and Korea, *E. japonicum* has demonstrated potent free radical scavenging abilities and anti-proliferative activities against breast cancer cells [91,92]. Prior research has investigated the anti-inflammatory efficacy of *E. japonicum* EE (EJEE) using an OVA-induced allergic asthma murine model [76]. This study revealed that oral EJEE administration (60 or 600 mg/kg) significantly suppressed the infiltration of immune cells—including eosinophils, neutrophils, lymphocytes, and monocytes—and attenuated the elevated IgE levels in the BALF. Furthermore, histological analyses via H&E and PAS staining showed that EJEE mitigated peribronchial inflammatory cell recruitment and mucus hypersecretion. EJEE also downregulated the populations of CD4+, CD8+, and CD19+ cells, suppressed GATA-3 expression, and inhibited the production of pro-inflammatory cytokines such as TNF-α and various interleukins (IL-4, 5, 6, and 13) in lung tissues. Notably, the therapeutic impact of 600 mg/kg EJEE was comparable to that of 1 mg/kg dexamethasone (DEX), which served as a positive control. From an extraction standpoint, EJEE was classified as a chemically characterized crude ethanol extract. Extraction yield, solvent ratio, extraction time, and temperature were recorded as NR when these parameters were not reported in the original study. Given the ethanol-based extraction, EJEE likely represents a broadly polar to intermediate-polarity preparation enriched in phenolic constituents rather than a lipophilic fraction. HPLC analysis identified chlorogenic acid and caffeic acid as major phenolic markers of EJEE. Because these compounds were not individually tested in the asthma model, they should be interpreted as candidate contributors and useful standardization markers rather than definitive active principles. Their known anti-inflammatory and antioxidant properties provide a plausible chemical basis for the observed suppression of Th2 cytokines, GATA3 expression, IgE production, and mucus hypersecretion.

### 6.4. Salvia plebeia

*Salvia plebeia* is a biennial herb that is widely distributed across various regions. This plant is well-regarded for its antioxidant and -inflammatory properties [93]. A previous study explored the immunomodulatory potential of *S. plebeian* aerial parts (SPAP) EE [SPAPEE] and *S. plebeian* root (SPR) EE [SPREE] using both in vitro and in vivo asthma models [54]. SPAPEE and SPREE were classified as crude ethanol extracts prepared from aerial parts and roots, respectively. Extraction yield, solvent concentration, and extraction conditions were recorded as NR when unavailable in the original article. Because the study compared aerial and root ethanol extracts, it provides an opportunity to discuss how plant part and extraction solvent may influence anti-inflammatory potency. The researchers demonstrated that both SPAPEE and SPREE effectively suppressed the LPS-induced production of NO, TNF-α, and IL-6 in RAW 264.7 macrophages. Notably, the inhibitory effect of 1000 μg/mL SPAPEE on NO and IL-6 was comparable to that of 1 μg/mL DEX. Furthermore, both extracts attenuated IL-6 and IL-8 upregulation in stimulated BEAS-2B cells, with 1000 μg/mL SPAPEE showing superior IL-6 inhibition compared to 100 μg/mL DEX. In an OVA-induced murine asthma model, oral administration of 100 mg/kg SPAPEE successfully reduced airway mucus production and eosinophil, neutrophil, and Th2 cytokine (IL-4, IL-5, and IL-13) levels in the BALF. These in vivo effects of 100 mg/kg SPAPEE were found to be equivalent to the outcomes observed with 3 mg/kg DEX.

### 6.5. Rosae multiflorae Fructus

Indigenous to East Asian regions, including Korea, Japan, and China, *R. multiflorae* has been traditionally employed as a therapeutic remedy for its antipyretic, detoxifying, and diuretic properties [94]. Song et al. reported that oral treatment with *R. multiflorae* fruit extract (FE) [RMFE] effectively mitigated airway inflammation in an OVA-induced murine model by leading to a significant reduction in lymphocyte and eosinophil counts within the lungs and alleviated pathological features, including goblet cell hyperplasia, mucus overproduction, collagen deposition, and eosinophilic infiltration [69]. Furthermore, RMFE administration significantly lowered pro-inflammatory and Th2-associated cytokine (TNF-α, IL-4, and IL-6) levels in the BALF. Notably, RMFE demonstrated a dose-dependent inhibitory effect on mast cell degranulation and histamine release triggered by compound 48/80.

### 6.6. Eclipta prostrata

In Brazilian traditional medicine, *E. prostrata* has long been utilized for managing asthma and various respiratory ailments [77]. A prior study investigated the therapeutic efficacy of *E. prostrata* methanol extract (ME) [EPME] in an OVA-sensitized BALB/c mouse model [77]. The findings demonstrated that the intranasal administration of 500 mg/kg EPME significantly reduced airway resistance and inhibited eosinophil infiltration, as well as IL-13 upregulation. Furthermore, phytochemical profiling via quantitative analysis identified wedelolactone, demethylwedelolactone, and oroboside as EPME’s primary bioactive constituents. Wedelolactone and demethylwedelolactone belong to the coumestan-type polyphenolic class, whereas oroboside is a flavonoid glycoside. These constituents may contribute to the suppression of IL-13-driven eosinophilic inflammation; however, direct compound-level validation in the same asthma model remains necessary.

### 6.7. Cnidium monnieri

Primarily distributed throughout East Asia, including Korea, China, and Japan, *C. monnieri* has been traditionally valued for its extensive pharmacological benefits, particularly its anti-inflammatory, anti-allergic, and skin-protective properties [95]. To investigate its medicinal value, Wang et al. tested the major component of *C. monnieri* fruit (CMF), osthole, in an OVA-induced asthma model [78]. Their results showed that it (25, 50, and 100 mg/kg) significantly attenuated the influx of eosinophils and other immune cells, while concurrently reducing Th2 cytokines and serum IgE. Osthole also improved mucus hypersecretion. Additionally, the study revealed that osthole hindered NF-κB nuclear translocation and IκB activation in lung tissues. The potency of 100 mg/kg osthole was equivalent to 2 mg/kg of DEX, confirming its potential as a treatment for allergic asthma.

### 6.8. Bupleurum chinense

A prominent species within the *Bupleurum* genus, *B. chinense,* has been a staple of traditional herbal medicine for over a millennium [96]. Despite its long history, the specific anti-inflammatory impact of *B. chinense* root extract (RE) [BCRE] on asthmatic airway inflammation has remained poorly understood. Previous investigations demonstrated that BCRE promotes the differentiation of naive CD4+ T cells into Th1 and Tr1 phenotypes by stimulating IFN-γ and IL-10 production [79]. In a murine model of OVA-induced asthma, the oral administration of BCRE (200 mg/kg) significantly attenuated eosinophil, neutrophil, and macrophage recruitment and successfully mitigated lung tissue remodeling, including inflammatory cell infiltration, mucus hypersecretion, and collagen deposition. Furthermore, BCRE suppressed the elevated levels of Th2/Th17-related cytokines and various inflammatory mediators, alongside reducing serum IgE/IgG1/IgG2a and inhibiting the NF-κB/IκBα signaling pathway in the lungs.

### 6.9. Artemisia pallens

*A. pallens* is extensively cultivated in regions such as Tamil Nadu in Southern India [80]. Studies have documented its significant pharmacological potential, specifically highlighting its anti-diabetic, -inflammatory, and -microbial activities. To explore its anti-asthmatic potential, *A. pallens* ME [APME] was tested in an OVA-challenged rat model [80]. APME treatment significantly lowered AHR and eosinophil and neutrophil counts. APME also provided potent antioxidant and -inflammatory effects, as evidenced by the modulation of SOD, GSH, MDA, and serum IgE levels. Moreover, APME curtailed the expression of pro-inflammatory cytokines and TGF-β while restoring Nrf2 mRNA levels in the lungs. Pathological improvements included reduced cell recruitment and lung fibrosis. Notably, APME exhibited a robust dose-dependent amelioration of asthmatic hallmarks across the 100, 200, and 400 mg/kg treatment groups. Specifically, the highest dose of 400 mg/kg attained a therapeutic efficacy comparable to that of montelukast (MON, 10 mg/kg), a conventional oral agent for asthma. This dose–response profile confirms that APME can reach a functional threshold equivalent to established reference standards, providing clear evidence of its pharmacological potency.

### 6.10. Artemisia argyi

*A. argyi* serves as a cornerstone of traditional East Asian medicine; it has been documented in Chinese pharmacopeias for over two millennia and remains a vital therapeutic agent in both Japanese (Kampo) and Korean traditional practices [81]. A study by Shin et al. established that the *A. argyi* ME (AAME) and its bioactive constituent, dehydromatricarin A (DA), possess potent anti-inflammatory properties against OVA-induced allergic airway inflammation [81]. Their findings revealed that treatment with 100 mg/kg AAME or 20 mg/kg DA effectively curtailed AHR, mucus hypersecretion, and neutrophil, macrophage, and eosinophil infiltration. Furthermore, these administrations suppressed ERK and MMP-9 activation while downregulating Th2 cytokine expression (IL-4, IL-5, and IL-13) and serum IgE levels.

### 6.11. Peucedanum japonicum

The roots of *P. japonicum* have a long history of use in Korean and Japanese traditional medicine for managing respiratory and inflammatory conditions, including headaches, colds, and coughs [55]. Chun et al. demonstrated that *P. japonicum* EE (PJEE) exhibits potent anti-inflammatory activity in LPS-stimulated RAW 264.7 macrophages at concentrations of 200 and 400 μg/mL [55]. These effects were mediated by the suppression of pro-inflammatory mediators such as NO, PGE2, TNF-α, and IL-6, as well as the downregulation of iNOS and COX-2 expression. Furthermore, PJEE was found to inhibit the Th2 polarization of naive CD4+ splenocytes in vitro. In an in vivo murine model of asthma, oral administration of 200 mg/kg PJEE significantly mitigated airway inflammation by regulating leukocyte infiltration, mucus hypersecretion, and Th2-associated factors, including GATA3 expression and IL-4/5/13 production. Notably, the anti-asthmatic efficacy of 200 mg/kg PJEE was comparable to that of 10 mg/kg MON. Phytochemical analysis identified peujaponiside, pteryxin, hyuganin C, and several peucedanol derivatives as PJEE’s primary constituents. These compounds, particularly pteryxin and peucedanol derivatives, represent coumarin-related phytochemical markers of PJEE. Their inclusion strengthens the chemical standardization of the extract, but the relative contribution of each constituent to GATA3 and Th2 cytokine inhibition should be clarified in future fractionation or isolated-compound studies.

### 6.12. Anthriscus sylvestris

A perennial herb native to Asia, *A. sylvestris* has traditionally been used to manage respiratory ailments such as bronchitis and persistent cough [97]. Kim et al. investigated the therapeutic potential of *A. sylvestris* RE (ASRE) against pulmonary inflammation using both in vitro and in vivo asthma models [82]. Their findings revealed that ASRE (100–200 µg/mL) effectively suppressed Th2 cell activation and IL-5 production in vitro. Additionally, ASRE (125–500 µg/mL) inhibited NO and IL-6 secretion in LPS-stimulated RAW 264.7 macrophages. In OVA-exposed C7BL/6 mice, oral administration of 200 mg/kg ASRE significantly ameliorated asthmatic features, including eosinophil recruitment, mucus hypersecretion, and Th2 cytokine (IL-4, IL-5, and IL-13), IgE, and eotaxin-3 elevation in the BALF. Furthermore, ASRE treatment downregulated GATA3, iNOS, and IRF4 expression, confirming its ability to modulate Th2-mediated allergic responses.

### 6.13. Hyssopus cuspidatus

*H. cuspidatus* has a long-standing history in Uighur medicine for treating bronchial asthma [56]; however, its specific impact on bronchitis has remained largely unexplored. Fengjuan et al. demonstrated that *H. cuspidatus* EE (HCEE) significantly curtailed NO, TNF-α, IL-6, and ROS production in LPS-activated RAW 264.7 cells at concentrations of 10 and 50 μg/mL [56]. These in vitro anti-inflammatory actions were mediated through MAPK and NF-κB signaling pathway inhibition. Furthermore, the researchers reported that HCEE effectively alleviates bronchitis in an OVA-induced asthmatic rat model by reducing serum IgE levels, blood eosinophil counts, and pro-inflammatory cytokine (IL-4, IL-6, IL-17, and TNF-α) and eotaxin secretion in the BALF. Notably, the therapeutic efficacy of 100 mg/kg HCEE was comparable to that of 1.2 mg/kg DEX. Phytochemical analysis revealed that HCEE contains several bioactive markers, including rosmarinic acid, hyperoside, salvigenin, diosmin, and 3,4-dimethoxycinnamic acid. These markers include phenolic acids and flavonoid/flavone derivatives, suggesting that HCEE may act through a polyphenol-rich anti-inflammatory profile. The reported inhibition of MAPK and NF-κB signaling is consistent with this phytochemical composition, although direct target engagement by each compound was not demonstrated.

### 6.14. Physalis peruviana

*P. peruviana* is widely recognized for its diverse pharmacological benefits, particularly its potent antioxidant activity [98]. Park et al. investigated the anti-inflammatory potential of *P. peruviana* ME (PPME) using LPS-activated RAW 264.7 cells and an OVA-induced murine model of bronchitis [57]. The in vitro findings revealed that PPME (20 and 40 μg/mL) effectively suppressed LPS-induced MCP-1 secretion. In the in vivo model, the administration of 5 mg/kg PPME significantly attenuated macrophage and eosinophil recruitment, while lowering Th2 cytokine (IL-4, IL-5, and IL-13) levels in the BALF and systemic IgE. Furthermore, PPME downregulated MCP-1 and KEN-5 expression in lung tissues by modulating the p38, JNK, and NF-κB signaling pathways. Notably, the therapeutic outcomes of 5 mg/kg PPME were comparable to those of 1 mg/kg DEX.

### 6.15. Pistacia weinmannifolia

Previous pharmacological investigations have highlighted the therapeutic potential of *P. weinmannifolia* RE (PWRE), a traditional herbal remedy used in China for inflammatory conditions [63]. In cellular models, PWRE exhibited robust anti-inflammatory properties by significantly suppressing MCP-1 production in LPS-stimulated RAW264.7 macrophages, an effect closely associated with the potent inhibition of NF-κB signaling. Translating these findings to an in vivo context, PWRE administration in OVA-induced asthmatic mice resulted in a marked reduction in eosinophil infiltration and a decrease in Th2-associated cytokines (IL-4, IL-5, and IL-13) within the BALF. Furthermore, PWRE treatment effectively lowered systemic allergic markers, such as total and OVA-specific IgE levels in the serum, while simultaneously alleviating pulmonary histological changes, including inflammatory cell influx and goblet cell-mediated mucus hypersecretion. Notably, these systemic improvements were accompanied by the significant suppression of MCP-1 expression within the lung tissues. These inhibitory effects were mechanistically linked to the downregulation of critical signaling axes, specifically the MAPK and NF-κB pathways. Notably, the therapeutic efficacy of 15 mg/kg PWRE in the in vivo model was comparable to that of 30 mg/kg MON, a widely used leukotriene receptor antagonist for asthma management.

### 6.16. Eriobotrya japonica

The leaves of *E. japonica* have a well-established history in traditional medicine as potent anti-inflammatory and -tussive agents in managing bronchial ailments [99]. Previous research has investigated the therapeutic potential of *E. japonica* leaf extract (LE) [EJLE] using both tracheal smooth muscle (TSM) and RAW 264.7 cells, alongside murine asthma models [58]. In cellular assays, 250 μg/mL of EJLE effectively suppressed TNF-α-induced MMP-2 production, ERK phosphorylation, and NF-κB nuclear translocation in TSM cells. Additionally, it inhibited the expression of iNOS and COX-2 in LPS-stimulated RAW 264.7 macrophages. In vivo evaluations further demonstrated that oral administration of 200 mg/kg EJLE significantly alleviated asthmatic responses by reducing serum IgE levels and attenuating IL-4, IL-13, NO, and eosinophil peroxidase (EPO) elevation in the BALF.

### 6.17. Dryopteris crassirhizoma

*D. crassirhizoma* is extensively distributed throughout the temperate regions of East Asia, including Korea, Japan, and China [100]. This fern species is recognized for its broad spectrum of pharmacological properties, such as its antioxidant, -tumor, -bacterial, and -viral activities against influenza and reverse transcriptase [101]. A prior study investigated the anti-asthmatic potential of *D. crassirhizoma* EE (DCEE) using various experimental models [70]. In vitro assays demonstrated that DCEE (0.1–10 mg/mL) effectively inhibited IL-6 and TNF-α secretion in PMA/A23187-stimulated human mast cells (HMCs). In an in vivo murine model of asthma, oral administration of 20 mg/kg DCEE significantly attenuated the elevation of inflammatory markers, including leukocyte infiltration (eosinophils and neutrophils), mucus hypersecretion, collagen deposition, and Th2-associated cytokines. Furthermore, DCEE suppressed systemic IgE/IgG1 responses and hindered NF-κB activation in lung tissues. Phytochemical characterization identified isoquercetin, chlorogenic acid, and pinellic acid as key bioactive constituents of DCEE. Isoquercetin and chlorogenic acid represent flavonoid and phenolic acid markers, respectively, whereas pinellic acid may reflect a lipid-derived bioactive component. These constituents provide a plausible chemical basis for mast-cell cytokine suppression and pulmonary NF-κB inhibition, but their individual contribution requires further validation.

### 6.18. Lindera obtusiloba

*L. obtusiloba* is a plant that is indigenous to Northeast Asia; it is traditionally utilized to enhance blood circulation and mitigate inflammatory conditions [102]. Previous research has explored the therapeutic potential of *L. obtusiloba* ME (LOME) against asthma [83]. In vitro experiments demonstrated that LOME exerts anti-inflammatory properties in TNF-α- stimulated H292 cells by downregulating the mRNA expression of Th2 cytokines (IL-4, IL-5, and IL-13) and suppressing IL-6 production, mediated through NF-κB signaling inhibition. In vivo studies further confirmed that oral LOME treatment significantly reduced airway AHR, inflammatory cell infiltration (macrophages and eosinophils), and various biomarkers, including MUC5AC, eotaxin, IgE, and oxidative stress indicators. Histopathological evidence showed a marked decrease in mucus overproduction and cellular influx. Mechanistically, LOME modulated the MAPK/AP-1/NF-κB pathways while enhancing the antioxidant defense via HO-1 and NQO1 upregulation. Notably, the efficacy of 100 mg/kg LOME was found to be comparable to 3 mg/kg of DEX. Quercetin and kaempferol rhamnosides were identified as major flavonoid glycoside markers of LOME and may contribute to its inhibition of MAPK/AP-1/NF-κB signaling and enhancement of antioxidant responses such as HO-1 and NQO1 induction.

### 6.19. Myxopyrum serratulum

*M. serratulum* has long been utilized in traditional Indian medicine to manage respiratory and inflammatory conditions, including cough and asthma [103]. Recent investigations into *M. serratulum* ME (MSME) have validated its anti-inflammatory properties, using both LPS-stimulated RAW264.7 cells and OVA-induced murine models [50]. More specifically, MSME (at 120 and 250 μg/mL) was found to significantly suppress the production of NO, ROS, and an array of pro-inflammatory cytokines such as TNF-α, IL-6, and various interleukins. In animal models, an oral dose of 400 mg/kg MSME exhibited therapeutic efficacy comparable to 3 mg/kg DEX, effectively reducing AHR, inflammatory cell infiltration, and the levels of mediators like PGE2 and Th2 cytokines. Furthermore, histopathological and protein analysis confirmed that MEMS mitigates mucus hypersecretion and downregulates iNOS and COX-2 expression within the lung tissues of asthmatic mice. Through HPLC characterization, p-coumaric acid, catechin, and naringenin were confirmed as the primary bioactive compounds within MSME. These markers represent phenolic acid, flavanol, and flavanone classes, respectively. Their co-occurrence suggests that MSME may exert anti-asthmatic effects through combined antioxidant, NF-κB/MAPK-modulating, and cytokine-suppressive mechanisms.

### 6.20. Scrophularia koraiensis

*S. koraiensis* is a native Korean medicinal herb traditionally employed to alleviate conditions such as fever and edema [59]. Jung et al. investigated the therapeutic potential of *S. koraiensis* EE (SKEE) in a murine model of experimental asthma [59]. Their findings revealed that oral administration of 40 mg/kg SKEE effectively mitigated ovalbumin-induced bronchitis. This improvement was characterized by AHR suppression, a reduction in eosinophil and neutrophil counts, and lower IL-5 and IL-13 levels in the BALF. Furthermore, SKEE treatment led to a decrease in serum IgE and inhibited pulmonary mucus hypersecretion. Mechanistically, 40 mg/kg of SKEE attenuated NF-κB activation and iNOS expression while promoting HO-1 induction in the lung tissues. Aucubin and harpagide, two iridoid glycosides, were identified as major phytochemical markers of SKEE and may contribute to NF-κB inhibition, iNOS suppression, and HO-1 induction.

### 6.21. Sophora japonica

Native to East Asia, particularly China and Korea, *S. japonica* is highly valued in traditional medicine for its hemostatic and anti-inflammatory properties [104]. A study by Kim et al. revealed that sophoricoside, a bioactive compound isolated from *S. japonica*, acts as an immune modulator in both in vitro and in vivo asthma models [71]. Their findings indicated that treatment with 30 μM sophoricoside effectively suppressed the production of IgE/antigen-induced mediators, such as PGD2, LTB4, and LTC4, in HMC-1 mast cells. Additionally, the compound was found to inhibit CD4+ T cell differentiation. In animal experiments, oral administration of 30 mg/kg sophoricoside led to a marked alleviation of asthmatic symptoms. This therapeutic effect was evidenced by a reduction in nasal rubbing, a decrease in inflammatory cell infiltration (including macrophages, eosinophils, neutrophils, and lymphocytes), and the downregulation of various cytokines (IFN-γ, TNF-α, and IL-4/5/13/17) as well as IgE, IgG1, IgG2a, histamine, and LTC4.

### 6.22. Angelica reflexa

In the traditional medicine of Northeast Asia, the roots of *A. reflexa* have long been prescribed to alleviate respiratory issues, such as cough and phlegm, as well as painful conditions, including neuralgia and arthralgia [72]. A recent study investigated the therapeutic potential of *A. reflexa* EE (AREE) in an experimental asthma model [72]. The in vitro results revealed that AREE (at concentrations of 125, 250, and 500 μg/mL) effectively suppressed NO, iNOS, and IL-6 production in activated RAW264.7 macrophages. In vivo experiments further demonstrated that oral administration of AREE (100 and 200 mg/kg) mitigated OVA-induced asthma symptoms in BALB/c mice. This was evidenced by a significant reduction in eosinophil and neutrophil infiltration, as well as decreased IL-5, IL-13, IgE, and eotaxin-3 levels in the BALF. Furthermore, AREE treatment inhibited Th2 cell activation, iNOS expression, and IRF4 signaling within the lung tissues. Histological evaluations confirmed that AREE effectively alleviated inflammatory cell accumulation around the airway epithelium and suppressed excessive mucus production.

### 6.23. Callicarpa japonica

*C. japonica* has historically served as an important herbal remedy across East Asia, specifically in terms of its clinical application in treating inflammation-related disorders [105]. A recent investigation by Kim et al. established the anti-inflammatory potential of *C. japonica* ME (CJME) in cellular models [48]. At concentrations of 40 and 80 μg/mL, CJME effectively suppressed LPS-induced TNF-α in RAW264.7 cells, while also inhibiting IL-6, IL-8, and MCP-1 production in PMA-stimulated A549 cells by blocking IκBα and NF-κB signaling. These findings were further validated in vivo, where oral administration of 40 mg/kg CJME significantly alleviated asthmatic symptoms. This therapeutic effect was characterized by a reduction in inflammatory cell infiltration (eosinophils and macrophages) and lower levels of Th2 cytokines (IL-4, IL-5 and IL-13), TNF-α and IL-6 in BALF, alongside decreased serum IgE and histamine. A histological and molecular analysis of lung tissues confirmed that CJME inhibited mucus hypersecretion and suppressed the activation of CREB and the NF-κB pathway. Notably, the efficacy of 40 mg/kg CJME was comparable to 1 mg/kg DEX. Additionally, CJME enhanced the expression of the antioxidant enzyme HO-1 across both models, with forsythoside B, verbascoside, and samioside identified as its primary bioactive constituents.

### 6.24. Alnus hirsuta

*A. hirsuta* is an East Asian medicinal plant traditionally utilized to manage conditions such as diarrhea and hemorrhage [106]. Lee and colleagues explored the anti-asthmatic potential of *A. hirsuta* ME (AHME) through both cellular and murine models [52]. Their in vitro results demonstrated that 80 μg/mL of AHME significantly reduced the TNF-α-induced expression of pro-inflammatory cytokines (IL-4, IL-5, IL-6, and IL-13) and MUC5AC in H292 cells by suppressing IκBα/NF-κB signaling. Consistent with these findings, in vivo experiments showed that 100 mg/kg AHME effectively reversed OVA-induced increases in airway resistance, AHR, and macrophage and eosinophil recruitment. Furthermore, AHME treatment led to a substantial decline in BALF cytokines, eotaxin, and serum IgE levels, while simultaneously alleviating pulmonary mucus hypersecretion. Oregonin, a diarylheptanoid glycoside, was identified as a major marker compound of AHME. Its presence provides a plausible chemical explanation for the observed inhibition of MAPK/NF-κB activation, although direct isolated-compound validation in asthma models remains warranted.

### 6.25. Castanea crenata

The inner shell of *C. crenata*, commonly known as chestnut, is rich in diverse polyphenolic compounds that possess significant biological activities [107]. A recent study by Kim et al. evaluated the therapeutic potential of *C. crenata* EE (CCEE) in a murine model of OVA-induced asthma [84]. Oral administration of 300 mg/kg CCEE led to a substantial reduction in AHR and inflammatory cell infiltration, specifically eosinophils and macrophages, in the BALF. Furthermore, CCEE treatment effectively lowered BALF cytokine levels (IL-4, IL-5, and IL-13) and serum IgE, while alleviating pulmonary mucus hypersecretion. At the molecular level, CCEE downregulated the elevated expression of iNOS, COX-2, and MMP-9, and inhibited NF-κB activation within the lung tissues. Ellagic acid, an ellagitannin-derived polyphenol, was identified as a major marker of CCEE. This compound may contribute to the suppression of iNOS, COX-2, MMP-9, and NF-κB signaling, but the extract-level activity should not be attributed exclusively to ellagic acid without further fractionation studies.

### 6.26. Phlomis umbrosa

*P. umbrosa* has a history of use as a traditional herbal remedy for managing diverse inflammatory diseases [108]. Building upon this background, Pak et al. examined the inhibitory effects of *P. umbrosa* EE (PUEE) on OVA-induced asthma in a murine model [85]. The experimental results indicated that oral administration of 40 mg/kg PUEE significantly inhibited the pronounced increase in immune cells (eosinophils, macrophages, and lymphocytes) and cytokines (IL-4, IL-5, and IL-13) within the BALF, as well as serum IgE levels in the OVA group. Furthermore, PUEE treatment was found to suppress AHR, the activation of the ERK/NF-κB pathways, and excessive mucus secretion in the lung tissues. The observed therapeutic efficacy of PUEE (40 mg/kg) was comparable to that of 30 mg/kg MON. Umbroside, shanzhiside methyl ester, and seamoside are iridoid glycoside-type constituents, suggesting that PUEE may represent an iridoid-rich extract with activity against ERK/NF-κB signaling, Th2 cytokine production, and mucus hypersecretion.

### 6.27. Artemisia gmelinii

In various East Asian countries, including Korea and China, *A. gmelinii* has long been utilized as a traditional herbal remedy for managing hepatobiliary disorders, such as hepatitis, jaundice, and cholecystitis [109]. In an experimental asthma model, a recent study demonstrated the protective properties of A. gmelinii extract (AGE) [73]. In vitro experiments revealed that AGE (5 μg/mL) effectively inhibited mast cell degranulation triggered by compound 48/80. In vivo, oral administration of 200 mg/kg AGE significantly mitigated asthmatic symptoms in BALB/c mice, which were characterized by alleviated inflammatory cell counts (macrophages, eosinophils, and neutrophils), mast cell infiltration, and reduced Th2-related cytokines (IL-4, IL-5, and IL-13) as well as IgE and histamine. Furthermore, AGE treatment was found to restore IL-12 levels and suppress pulmonary mucus hypersecretion. These therapeutic effects were primarily attributed to transcription factor modulation, specifically through T-bet upregulation and the concomitant GATA-3 downregulation.

### 6.28. Gynostemma pentaphyllum

Indigenous to several East and Southeast Asian nations, including Korea, Japan, and China, *G. pentaphyllum* is esteemed for its capacity to bolster metabolic functions and mitigate oxidative damage [110]. In experimental models of bronchitis, recent research has established the anti-asthmatic potential of gypenoside A (GA), a bioactive constituent isolated from *G. pentaphyllum* [64]. In vitro experiments revealed that GA effectively suppressed the OVA-induced secretion of Th2 cytokines (IL-4, IL-5, and IL-13) in splenocytes. Furthermore, GA treatment inhibited the production of various inflammatory mediators, including IL-6, IL-8, MCP-1, and several chemokines (CCL5, CCL11, CCL24), as well as ROS in IL-4/TNF-α-stimulated BEAS-2B cells. These findings were validated in vivo, where oral administration of 30 mg/kg GA markedly mitigated AHR, pulmonary mucus overproduction, and eosinophil and monocyte infiltration in asthmatic mice. Mechanistically, GA is a dammarane-type saponin and represents one of the clearest examples in this review in which a defined constituent, rather than only a crude extract, was evaluated across asthma-relevant endpoints. Its activity was associated with suppression of Th2 cytokines, chemokines, ROS, IgE/IgG1, MDA, and COX-2.

### 6.29. Adenophora stricta

Historically recognized within East Asia, the roots of *A. stricta* are frequently prescribed to manage respiratory discomfort, specifically because of its expectorant and antitussive properties [86]. A recent study evaluated the therapeutic potential of *A. stricta* aqueous extract (AE) [ASAE] in mitigating bronchitis [86]. In vitro experiments revealed that 3 mg/mL of AEAS significantly suppressed the production of pro-inflammatory mediators, including NO, TNF-α, IL-1β, IL-6, and MCP-1, as well as the mRNA expression of iNOS in LPS-activated RAW264.7 cells. These anti-inflammatory effects were mediated by the downregulation of LPS-induced JNK, NF-κB, and IκBα signaling pathways. In an in vivo model, OVA sensitization and inhalation were utilized to induce bronchitis in mice. AEAS administration effectively alleviated bronchial inflammation by reducing the elevated eosinophil, mast cell, IL-4, IL-5, and IgE levels characteristic of the OVA-exposed group.

### 6.30. Fritillaria unibracteata

As a key constituent of the traditional medicine *Fritillariae Cirrhosae Bulbus*, *F. unibracteata* is valued for its therapeutic properties [111]. Despite its traditional uses, the immunomodulatory influence of *F. unibracteata* total alkaloids (TA) [FUTA] on asthma pathogenesis has not been fully elucidated. To address this, Peng et al. conducted a comprehensive investigation using both in vitro and in vivo approaches [65]. Their findings revealed that FUTA treatment significantly downregulated TRPV1 and NFAT expression, while reducing TSLP production and suppressing p38 activation in TNF-α- or IL-4-stimulated BEAS-2B cells. In animal models, OVA-induced asthmatic mice exhibited marked increases in AHR, pulmonary mucus secretion, collagen deposition, and serum IgE. Furthermore, elevated levels of inflammatory mediators—including IL-1β, IL-4, IL-17A, IL-33, TNF-α, and CD45—were observed in the lungs of the OVA group. Notably, oral FUTA administration effectively mitigated these asthmatic phenotypes and attenuated the pulmonary expression of TRPV1, NFAT, TSLP, and phosphorylated p38. Phytochemical analysis identified peiminine, peimine, edpetiline, khasianine, peimisine, and sipeimine as FUTA’s primary bioactive constituents. These constituents are steroidal alkaloids characteristic of Fritillaria species. Their association with TRPV1/Ca^2+^/NFAT, TSLP, and p38 signaling suggests that Fritillaria alkaloids may be particularly relevant to epithelial-alarmin and neuroimmune inflammatory pathways in asthma.

### 6.31. Scrophularia takesimensis

*S. takesimensis* is a medicinal herb endemic to Ulleung Island, South Korea. Studies have highlighted its diverse pharmacological properties, particularly its potent antioxidant activity and cellular protective effects [74]. A recent investigation explored the anti-asthmatic potential of *S. takesimensis* EE (STEE) using an in vivo murine model [74]. The study found that oral treatment with 200 mg/kg STEE effectively mitigated several asthmatic indicators, including the elevation of eosinophils, Th2 cells, and IL-4 levels within the BALF. Furthermore, STEE administration led to a significant reduction in serum IgE and inhibited pulmonary mucus hypersecretion. These therapeutic benefits were attributed to Th2 cell activation suppression. Notably, the efficacy of 200 mg/kg STEE was found to be comparable to that of DEX.

### 6.32. Dictamnus dasycarpus

A member of the Rutaceae family indigenous to Korea, China, and Japan, *D. dasycarpus* has been recognized for its anti-atopic properties [112]; however, its potential efficacy against asthma has not been fully explored. Recently, Jung et al. investigated the therapeutic impact of *D. dasycarpus* AE [DDAE] on airway inflammation and mucus hypersecretion [66]. In vitro experiments using IL-4-/IL-13-stimulated human bronchial epithelial cells demonstrated that 100 μg/mL of DDAE significantly prevented MUC5AC upregulation and FoxA2 downregulation by suppressing the STAT3/STAT6 signaling pathway. In vivo results further showed that oral administration of DDAE (100 and 300 mg/kg) effectively reduced eosinophil infiltration and Th2 cytokine (IL-4, IL-5, and IL-13) and chemokine (TARC, MDC, and IP-10) production in the BALF of asthmatic mice. Additionally, DDAE treatment led to a decline in serum IgE levels and alleviated pulmonary mucus overproduction, fibrosis, and AHR. These improvements were associated with pulmonary FOXA2 modulation and STAT3/STAT6 activation inhibition. Phytochemical analysis identified rutaevin, dictamnine, limonin, obacunone, and fraxinellone as DDAE’s primary bioactive constituents. This profile includes limonoids and furoquinoline alkaloids, chemical classes that may contribute to STAT3/STAT6 suppression, FOXA2 restoration, and MUC5AC regulation. However, because the extract, rather than each purified constituent, was primarily evaluated, these metabolites should be regarded as candidate markers for future standardization.

### 6.33. Camellia sinensis

Indigenous to the subtropical and tropical landscapes of Southwestern China, India, and Southeast Asia, *C. sinensis* is esteemed for its extensive pharmacological profile, which includes its potent antioxidant properties, anti-carcinogenic activities, and promotion of cardiovascular wellness [113]. According to the recent findings of Pak et al., *C. sinensis* EE (CSEE) exhibits significant anti-asthmatic properties in murine models [87]. Oral administration of 100 mg/kg CSEE was shown to effectively reduce airway resistance, serum IgE, and the accumulation of Th2-related cytokines (IL-4, IL-5, and IL-13) and inflammatory cells, such as eosinophils and macrophages, in the BALF. Histopathological examinations revealed that CSEE treatment alleviated lung tissue damage, specifically inhibiting inflammatory cell infiltration and mucus overproduction. Furthermore, CSEE downregulated NF-κB and IκBα activation as well as MMP-9 expression in the lungs, suggesting that its therapeutic efficacy is mediated through the modulation of these key signaling pathways. Phytochemical profiling of CSEE identified caffeine alongside several major catechins, including (-)-epigallocatechin, (-)-epicatechin, (-)-epigallocatechin gallate, and (-)-epicatechin gallate. Catechins such as epigallocatechin gallate and epicatechin gallate are polyphenolic flavanols, whereas caffeine is a purine alkaloid. The catechin-rich profile of CSEE provides a plausible basis for NF-κB/IκBα inhibition, MMP-9 downregulation, and Th2-associated inflammation reduction.

### 6.34. Spenceria ramalana

A perennial herb within the Rosaceae family, indigenous to the high-altitude regions of China, *S. ramalana* has recently garnered significant attention for its potent anti-inflammatory activities validated through contemporary pharmacological studies [60]. In a recent investigation by Xia et al., *S. ramalana* EE (SREE) was evaluated for its therapeutic potential and underlying molecular actions in a rat model of asthma [60]. Administration of SREE at a dose of 500 mg/kg markedly reversed asthmatic hallmarks, including elevated lung W/D ratio, inflammatory cell (eosinophil and neutrophil) infiltration into the BALF, and a systemic rise in cytokines such as IL-4, IL-5, IL-13, and TNF-α. Furthermore, SREE treatment effectively mitigated lung permeability, fibrotic collagen deposition, and goblet cell-mediated mucus overproduction. At the molecular level, SREE prompted the epithelial integrity restoration by upregulating tight junction proteins (ZO-1, Occludin, and Claudin-1), while simultaneously suppressing the expression of remodeling markers like MMP-9 and α-SMA. Notably, the pharmacological efficacy of 500 mg/kg SREE was found to be comparable to that of the reference glucocorticoid, DEX (2 mg/kg).

### 6.35. Alstonia scholaris

Commonly referred to as the ‘Scholar’s Tree’ or ‘Devil’s Tree,’ *A. scholar* is a tropical evergreen species widely distributed across the Indian subcontinent, Southeast Asia, and Northern Australia [114]. A recent study by Tong et al. explored the therapeutic potential of TA derived from *A. scholaris* (ASTA) in a murine model of OVA-induced bronchitis [61]. Histopathological evaluations revealed that ASTA treatment markedly suppressed inflammatory cell infiltration and airway mucus hypersecretion. Beyond structural improvements, ASTA effectively reversed the OVA-induced biochemical imbalances in the lungs; specifically, it restored E-cadherin and IFN-γ levels while significantly attenuating the overexpression of MUC5AC, eotaxin, and a broad spectrum of Th2/Th17-related cytokines, including IL-4, IL-5, IL-6, IL-8, IL-13, IL-9, IL-17A, IL-25, IL-33, and TSLP. Furthermore, ASTA administration led to a substantial reduction in ST2 expression, systemic IgE, and key inflammatory mediators such as MCP-1 and cysteinyl leukotrienes (LTB4, LTC4, LTD4, and LTE4). Chemical profiling of ASTA identified several bioactive indole alkaloids—including scholaricine, 19-epischolaricine, vallesamine, and picrinine—as the primary constituents responsible for these multifaceted anti-asthmatic activities. Scholaricine, 19-epischolaricine, vallesamine, and picrinine are indole alkaloids. Because ASTA was evaluated as a total alkaloid fraction, this study provides stronger class-level evidence than crude extract studies and suggests that indole alkaloid-rich fractions may regulate Th2/Th17 cytokines, epithelial mucus markers, IgE, ST2, and leukotriene-associated inflammatory mediators.

### 6.36. Melia azedarach

Widely recognized as the ‘Chinaberry tree’ or ‘Persian lilac,’ *M. azedarach* is an adaptable deciduous species indigenous to the tropical and subtropical regions of Asia and northern Australia [67]. A recent in vivo study demonstrated the potent immunomodulatory effects of *M. azedarach* EE (MAEE) in a BALB/c mouse model of OVA-induced bronchitis [67]. Asthma induction led to a significant elevation in AHR, extensive eosinophil recruitment, and increased levels of Th2 cytokines (IL-4, IL-5, and IL-13) in the BALF, alongside elevated systemic IgE. Oral administration of MAEE at 100 mg/kg markedly attenuated these pathological markers. Histopathological analysis further confirmed that MAEE treatment effectively mitigated inflammatory cell infiltration and airway mucus overproduction. Notably, MAEE enhanced the pulmonary antioxidant defense by upregulating HO-1 and SOD2, while simultaneously reducing oxidative stress markers such as 8-hydroxydeoxyguanosine (8-OHdG) and the remodeling enzyme MMP-9. Toosendanin, a limonoid-type triterpenoid, was identified as a major marker of MAEE and may contribute to its dual suppression of inflammatory and oxidative stress-related pathways, including Th2 cytokines, IgE, 8-OHdG, and MMP-9.

### 6.37. Hyssopus cuspidatus

Commonly known as Shen Xiang Cao, *H. cuspidatus* originates in Central Asia and Northwest China; it is highly regarded for its versatile roles in both medicine and cooking [115]. Recent in vivo research on asthma has highlighted the immunomodulatory potential of *H. cuspidatus* EE (HCEE) [88]. This study found that HCEE effectively mitigated AHR and reduced the accumulation of inflammatory cells, such as eosinophils and neutrophils, in both blood and BALF. Furthermore, HCEE administration suppressed the elevated levels of IgE and Th2-associated cytokines (IL-4, IL-5, and IL-13) while modulating the PI3K, JNK, and p38 signaling pathways within lung tissues. Phytochemical analysis revealed that HCEE contains diverse terpenoids, flavonoids, and phenolic acids. This mixed phytochemical profile suggests that the extract may act through coordinated inhibition of PI3K, JNK, and p38 signaling, but the specific metabolites responsible for these effects remain to be defined.

### 6.38. Shuteria involucrate

Although the roots of *S. involucrata*—a traditional Dai medicinal herb called ‘Tong-qian-ma-huang’—are widely recognized for their clinical effectiveness in treating asthma, the precise pharmacological mechanisms underlying its respiratory benefits have yet to be fully elucidated [116]. Bao et al. explored the anti-inflammatory potential of *S. involucrate* EE (SIEE) using activated THP-1 cells. The results demonstrated that a 200 μg/mL dosage effectively lowered LPS-induced levels of IL-6, IL-1β, IL-18, and MCP-1 by suppressing the TLR4/NF-κB signaling pathway [62]. In a subsequent in vivo assessment using an OVA-induced bronchitis model in BALB/c mice, the administration of 500 mg/kg SIEE significantly mitigated enhanced pause (Penh), mucus overproduction in the lungs, and elevated levels of MUC5AC and serum IgE. Furthermore, SIEE treatment led to a marked reduction in various inflammatory mediators in the BALF, including eotaxin and multiple Th2 cytokines (IL-4, IL-5, IL-9, and IL-13) while simultaneously inhibiting TLR4/NF-κB activation within the pulmonary tissues of asthmatic mice.

### 6.39. Inula japonica

Indigenous to East Asia, *I. japonica* maintains a broad geographic presence spanning the Korean Peninsula, China, Japan, and the Russian Far East [117]. A recent study validated the therapeutic potential of *Inula japonica* AE (IJAE) and its bioactive constituents—namely britannilactone, 6-methoxyluteolin, and 1-O-acetylbritannilactone—in both cellular and animal models of asthma [68]. IJAE was classified as an extract-plus-isolated-constituent study. The extraction solvent and yield of IJAE were summarized where available, and the isolated constituents were listed separately from the parent extract. This distinction is important because the extract represents a chemically complex preparation, whereas britannilactone, 6-methoxyluteolin, and 1-*O*-acetylbritannilactone represent defined constituents with direct pharmacological validation. At a concentration of 100 μg/mL, IJAE effectively suppressed JAK2, STAT3, and STAT6 phosphorylation in human bronchial epithelial cells stimulated with IL-4 and IL-13. Similarly, at 50 μg/mL, its active compounds exhibited potent inhibitory activities against the same signaling pathways. Furthermore, the in vivo administration of IJAE (100 and 300 mg/kg) in asthmatic mice significantly attenuated airway mucus hypersecretion and the pathological surge of IgE, periostin, and Th2-related mediators (IL-4, IL-5, IL-13, MDC, and eotaxin), while reducing the infiltration of inflammatory cells, including eosinophils, macrophages, and neutrophils. Britannilactone and 1-O-acetylbritannilactone are sesquiterpene lactones, whereas 6-methoxyluteolin is a flavone. This study is particularly valuable because both the extract and defined constituents were tested, linking these metabolites directly to inhibition of JAK2/STAT3/STAT6 signaling in bronchial epithelial cells.

### 6.40. Phellodendron amurense

Native to the Korean Peninsula, Northern China, Japan, and the Russian Far East, P. amurense is a significant botanical species whose trunk bark has long been prized as a core therapeutic agent in traditional Eastern medicine [118]. A recent investigation by Kim et al. explored the immunomodulatory potential of Phellodendri Cortex (PC), the desiccated trunk bark of *P. amurense*, using a murine model of OVA-induced asthma [89]. Their findings revealed that oral administration of the PC ME (PCME) effectively countered Penh, excessive mucus production, and subepithelial fibrosis, while reducing eosinophil infiltration and the expression of Th2-related cytokines and chemokines, including IL-4, IL-5, IL-13, TNF-α, IgE, CCL3, and TARC. Notably, the therapeutic efficacy of 100 mg/kg PCME was comparable to that of cyclosporine (10 mg/kg), a potent immunosuppressant typically reserved for severe steroid-dependent asthma. Phytochemical profiling of PCME identified several key bioactive alkaloids and limonoids, such as phellodendrine, jatrorrhizine, palmatine, berberine, and limonin. PCME should be annotated as a chemically characterized crude methanol extract of trunk bark. Because methanol extracts are broadly polar organic preparations, PCME was annotated as a chemically characterized crude methanol extract of trunk bark. Because methanol extracts are broadly polar organic preparations, PCME was not interpreted as a purified alkaloid fraction unless fractionation or enrichment was performed. Extraction yield, solvent concentration, and extraction conditions were recorded as NR when unavailable. 

## 7. Conclusions

This scoping review synthesized preclinical evidence published between 2005 and 2025, with a particular focus on the quantitative analysis of the past decade (2016–2025), to evaluate the anti-asthmatic potential of botanical extracts and phytochemical-rich fractions within established in vitro and in vivo systems. Our analytical scope specifically prioritized studies reporting asthma-relevant endpoints—including Th2 cytokines, IgE, airway hyperresponsiveness, and remodeling markers—while emphasizing mechanistic insights into the NF-κB, MAPK, and STAT3/6 signaling axes [119,120]. In contrast to earlier reviews that primarily catalogued natural products, phytochemicals, or herbal compounds with anti-asthmatic activity, this scoping review advances a distinct analytical framework by comparing botanical extracts according to plant-part source, experimental model, inflammatory stimulus, and mechanistic endpoint. This distinction is important because similar anti-inflammatory outcomes may arise from different extract types and model systems, thereby influencing reproducibility, mechanistic interpretation, and translational priority.

Quantitative stratification of the tested materials revealed a clear imbalance in the current evidence base. Among the literature reviewed for quantitative synthesis (2016–2025), 32 studies, or 82.1%, primarily evaluated crude botanical extracts. Of these, 15 studies lacked reported phytochemical markers or chromatographic characterization, whereas 17 studies provided some degree of chemical profiling or marker identification. Only 2 studies evaluated enriched fractions, 3 studies tested isolated compounds alone, and 2 studies examined both extracts and isolated constituents. Importantly, most chemically profiled extracts should be regarded as characterized rather than fully standardized, because quantitative marker specifications and batch-to-batch reproducibility were rarely reported. This stratification shows that the field remains dominated by crude or partially characterized preparations, limiting reproducibility, dose translation, and mechanistic interpretation. In addition to differences in material type, extraction-related reporting was highly variable across the reviewed studies. Although plant part information was usually available, extraction yield, solvent ratio, extraction duration, temperature, and fractionation procedures were often incompletely described. This limits reproducibility because ethanol, methanol, water, and non-polar or semi-polar solvents can enrich different chemical classes [121,122,123] and may therefore lead to different anti-asthmatic mechanisms even when the same plant species is used.

Across the 39 studies analyzed, several recurring mechanistic patterns emerged. First, the most consistent improvements were observed in IL-4, IL-5, IL-13, serum IgE, eosinophil infiltration, and mucus hypersecretion, indicating that most botanical interventions primarily target Th2-dominant allergic inflammation. Notably, a comprehensive analysis of these studies reveals that various botanical extracts derived from both aerial and underground organs showed directionally comparable effects to reference drugs, such as dexamethasone (DEX), methylprednisolone (MP), and montelukast (MON), within specific preclinical settings. Furthermore, although any mechanistic comparison with anti-IgE biologics remains premature, the reduced serum or free IgE observed in selected studies suggests possible upstream modulation of allergic sensitization pathways.

Second, while NF-κB and MAPK emerged as the most frequently modulated pathways, STAT3/6, GATA3, TSLP, and oxidative stress-related mechanisms were highlighted as increasingly important targets. From a phytochemical perspective, the reviewed evidence suggests that phenolic acids, flavonoids, catechins, coumarins, iridoid glycosides, alkaloids, limonoids, saponins, and sesquiterpene lactones are the most frequently reported secondary-metabolite classes in anti-asthmatic botanical preparations. Among these, polyphenol-rich extracts were most consistently associated with inhibition of NF-κB/MAPK signaling, oxidative stress, cytokine release, and epithelial mucus production. In contrast, alkaloid-, limonoid-, and saponin-rich preparations were more frequently linked to broader immunomodulatory effects involving Th2/Th17 cytokines, IgE, TSLP, TRPV1/NFAT, STAT3/6, and leukotriene-related mediators. Nevertheless, many studies identified phytochemical markers without directly testing the isolated compounds; therefore, causal attribution should remain cautious until activity-guided fractionation and compound-level validation are performed.

Third, an intriguing functional distinction was noted between plant organs: aboveground materials (leaves, flowers, and fruits) were frequently associated with epithelial protection and antioxidant activity, whereas underground organs (roots and rhizomes) more commonly demonstrated systemic immunomodulation and attenuation of Th2/IgE pathways. These patterns suggest a convergent model in which botanical extracts act through the coordinated suppression of epithelial activation, immune amplification, and remodeling-associated signaling.

Despite their utility in mechanistic screening, these cellular systems (e.g., RAW264.7, NCI-H292, and A549) and OVA-induced models should not be interpreted as full surrogates of human asthma. Transformed cell lines and specific inflammatory stimuli (e.g., LPS, IL-4/IL-13) model isolated pathways but incompletely reflect the immunological heterogeneity, epithelial barrier integrity, and temporal complexity of the disease in humans. While the OVA model robustly reproduces hallmark allergic features, it lacks the chronic remodeling and steroid-resistant phenotypes seen in patients. Therefore, these platforms are best regarded as reductionist systems for prioritizing extracts and generating mechanistic hypotheses for validation in more integrated, clinically relevant models.

To facilitate the translation of preclinical findings into clinical applications, future research should move beyond crude or incompletely characterized botanical preparations and prioritize chemically standardized extracts, bioactive-enriched fractions, and validated isolated constituents. The quantitative analysis in this review indicates that crude extract-based studies still dominate the current evidence base, whereas investigations using standardized extracts, enriched fractions, or isolated compounds remain limited. Therefore, future studies should provide quantitative phytochemical markers, chromatographic fingerprints, marker-based batch reproducibility data, and correlations between chemical profiles and biological activity. Future comparative studies should therefore report extraction solvent, solvent concentration, extraction yield, extraction time, extraction temperature, solid-to-liquid ratio, and fractionation workflow as core methodological variables. Without these details, biological activity cannot be reliably attributed to a reproducible botanical preparation, and cross-study comparison remains limited.

Further research should also aim to identify fraction- or constituent-specific molecular targets with greater precision. This approach is essential for reducing inter-study variability and improving the reproducibility and robustness of pharmacological findings. In addition, the strategic development of Korean herbal medicine-based asthma therapeutics will require more sophisticated experimental models that better reflect the clinical heterogeneity, chronic inflammation, and structural airway remodeling observed in human asthma. An integrated framework that standardizes extraction methods, chemical characterization, and mechanism-based validation will be critical for advancing botanical candidates toward clinically meaningful applications.

## 8. Future Directions

Despite the promising preclinical data identified in this scoping review, several translational hurdles must be cleared before botanical extracts can be effectively integrated into clinical asthma management. First, there is a critical need for phytochemical standardization. Our quantitative synthesis of literature from 2016–2025 shows that 32 of the 39 reviewed studies relied primarily on crude extracts, and 15 studies did not report specific phytochemical markers or chromatographic characterization. This limits dose translation, reproducibility, and mechanistic interpretation across studies. Second, while improvements in cytokines and histological markers are encouraging, more rigorous data on pharmacokinetics (PK), bioavailability, and long-term toxicological safety are required for most candidates. Third, the significant methodological heterogeneity in extraction protocols and dosing regimens makes it difficult to establish a unified efficacy profile across the literature. Finally, a fundamental limitation of current evidence is the over-reliance on acute OVA-induced models, which primarily replicate Th2-high allergic inflammation but fail to capture the profound immunological heterogeneity and persistent structural airway remodeling characteristic of human asthma.

Future research should therefore prioritize the following:

1. Establishing standardized bioactive fractions based on quantified phytochemical markers, followed by activity-guided fractionation and isolated-compound validation to confirm which metabolites are responsible for modulation of NF-κB, MAPK, STAT3/6, GATA3, TSLP, MUC5AC, IgE, and Th2/Th17 cytokine pathways;
2. Elucidating the dynamic processing of cytokine networks—specifically the systemic and local modulation of the IL-4/IL-5/IL-13 cascade—to better understand how botanical interventions influence the kinetic transition of the immune response;
3. Conducting longitudinal assessments of structural tissue modifications, such as goblet cell hyperplasia and subepithelial collagen deposition, to evaluate the potential of extracts in arresting chronic airway remodeling;
4. Conducting mechanism-guided comparisons across distinct asthma phenotypes (e.g., T2-low, neutrophilic);
5. Utilizing more clinically relevant platforms, such as human airway epithelial co-cultures and diverse allergen-exposure models;
6. Conducting extensive toxicological safety evaluations to establish comprehensive profiles of potential adverse effects and allergenic cross-reactivity. This is particularly crucial for individuals with pre-existing hypersensitivities, who should exercise caution until formal clinical safety guidelines and contraindications are clearly defined.

To address methodological inconsistencies between studies, we propose a standardized extraction-reporting framework for botanical asthma research. Future investigations should explicitly report: (i) raw material authentication, including botanical origin, plant part, harvest information, and voucher specimen; (ii) extraction solvent, solvent concentration, solvent polarity, and solid-to-liquid ratio; (iii) extraction yield expressed as percentage yield or mg extract per g dried material; (iv) extraction parameters such as temperature, pressure, duration, number of extraction cycles, and drying method; (v) whether the preparation represents a hydrophilic extract, hydroalcoholic/intermediate-polarity extract, lipophilic fraction, crude extract, partially purified fraction, enriched fraction, or isolated compound; (vi) phytochemical fingerprinting by HPLC, UPLC, LC-MS/MS, GC-MS, or equivalent methods; and (vii) transparent fractionation and purification procedures when enriched fractions or isolated constituents are tested. In addition, future reports should distinguish between compounds that are merely detected as extract markers and compounds that have been directly validated as active anti-asthmatic constituents. Quantitative reporting of marker content, batch-to-batch variability, and chemical-activity correlations will be essential for transforming crude botanical extracts into reproducible therapeutic candidates. Adopting this unified reporting standard will facilitate direct cross-study comparisons and enhance the pharmacological reproducibility of botanical asthma therapies.

Future reports should explicitly classify the tested material as an uncharacterized crude extract, chemically characterized crude extract, standardized extract, enriched fraction, or isolated compound. For crude extracts, authors should state whether phytochemical markers were detected and whether they were quantified. For standardized extracts, quantitative marker specifications and batch-to-batch reproducibility should be provided. For enriched fractions, the enrichment procedure, marker content, and chemical class specificity should be described. For isolated compounds, purity, structural confirmation, and dose equivalence to the parent extract should be reported. Such reporting will allow future reviews and meta-analyses to compare not only biological outcomes, but also the chemical reliability of the tested interventions. We further recommend that future botanical asthma studies include a minimum extraction-reporting template: plant species and voucher number; plant part; drying and pulverization method; extraction solvent and concentration; extraction temperature and time; solid-to-liquid ratio; extraction yield; fractionation method, if applicable; polarity or fraction classification; phytochemical marker identity and quantity; chromatographic fingerprint; and batch information. Incorporating these variables will improve reproducibility, enable dose normalization across studies, and clarify whether observed biological effects are associated with hydrophilic phenolics and glycosides, intermediate-polarity polyphenols and alkaloids, or more lipophilic terpenoids, limonoids, and essential-oil constituents.

This approach will help distinguish which botanical candidates are best suited as supportive nutraceuticals and which possess the therapeutic rigor necessary for formal drug development.

## Figures and Tables

**Table 1:** Quantitative Synthesis of Experimental Models and Key Parameters in Asthma Research.

Category	Experimental Mode	Usage (*n*) /Percentage (%)	Key Parameters /Endpoints	Clinical Relevance	References
In Vitro	RAW 264.7 (Macrophage)	55 (47%)	NO, iNOS, TNF-α, IL-6, NF-κB	Systemic inflammatory response & macrophage activation mimicry	[45,48,49,50,54,55,56,57,58,59,60,61,62]
	A549/H292 (Epithelial)	51 (44%)	MUC5AC, ROS, JAK-STAT, TSLP	Mucus hypersecretion & Airway injury	[49,53,57,63,64,65,66,67,68]
	Other Primary/Cell lines	12 (10%)	PI3K/Akt, NFAT, Cell viability	Supplementary mechanism profiling	[12,55,65,69,70,71,72,73,74]
In Vivo	OVA-induced (Mouse/Rat)	89 (76%)	IgE, AHR, Th2 cytokines, BALF, Histology	Allergic asthma & Type-2 response	[45,48,50,52,53,54,55,56,57,58,59,60,61,62,63,64,65,66,67,68,69,70,71,72,73,74,75,76,77,78,79,80,81,82,83,84,85,86,87,88,89]
	Other (HDM, etc.)	10 (9%)	Airway remodeling, Eosinophilic inflammation	Chronic/Clinically relevant exposure	[51,68]

Note: Percentages are based on a subset of the 120 cited references (*n* = 95) that specifically employed experimental asthma models. Percentages total > 100% as many studies utilized integrated in vitro and in vivo platforms simultaneously. Source: Compiled by authors based on a systematic analysis and quantitative synthesis of the cited literature (Refs. [1,2,3,4,5,6,7,8,9,10,11,12,13,14,15,16,17,18,19,20,21,22,23,24,25,26,27,28,29,30,31,32,33,34,35,36,37,38,39,40,41,42,43,44,45,46,47,48,49,50,51,52,53,54,55,56,57,58,59,60,61,62,63,64,65,66,67,68,69,70,71,72,73,74,75,76,77,78,79,80,81,82,83,84,85,86,87,88,89,90,91,92,93,94,95,96,97,98,99,100,101,102,103,104,105,106,107,108,109,110,111,112,113,114,115,116,117,118,119,120]).

**Table 2:** Numerical Synthesis of Molecular Mechanisms and Therapeutic Targets.

Category	Molecular Pathway/Target	Usage (*n*) /Percentage (%)	Primary Role in Asthma Research	References
Signaling	NF-κB/MAPK	80 (68%)	Pro-inflammatory transcriptional control	[38,44,45,48,49,50,53,54,55,56,57,58,59,62,64,65,68,70,71,73,79,84,85,86,87,88]
Immune	Th2 cytokines (IL-4, 5, 13)	91 (78%)	Essential allergic asthma pathology	[39,40,50,52,53,54,55,56,57,58,59,60,61,62,63,64,66,67,68,69,70,71,72,73,74,75,77,79,80,81,82,83,84,85,86,87,88,89]
Pathology	MUC5AC/Mucus pathways	44 (38%)	Mucus hypersecretion & obstruction	[49,50,52,53,57,60,63,64,65,66,67,68,86,116]
JAK-STAT	STAT6/STAT3/STAT1	41 (35%)	Th2 differentiation & Goblet cell hyperplasia	[39,40,65,66,68,116]
Advanced	PI3K/Akt/TRPV1/Nrf2	21 (18%)	Ca^2+^ signaling & Oxidative stress defense	[45,48,59,60,61,64,65,66,83,88]
Allergy	IgE/Mast cell activation	64 (55%)	Allergic sensitization & Histamine release	[19,49,50,52,53,55,69,70,71,73,89]

Note: Percentages are based on a subset of the 120 cited references (*n* = 95) that specifically employed experimental asthma models. Percentages total > 100% as many studies utilized integrated in vitro and in vivo platforms simultaneously. Source: Compiled by authors based on a systematic analysis and quantitative synthesis of the cited literature (Refs. [1,2,3,4,5,6,7,8,9,10,11,12,13,14,15,16,17,18,19,20,21,22,23,24,25,26,27,28,29,30,31,32,33,34,35,36,37,38,39,40,41,42,43,44,45,46,47,48,49,50,51,52,53,54,55,56,57,58,59,60,61,62,63,64,65,66,67,68,69,70,71,72,73,74,75,76,77,78,79,80,81,82,83,84,85,86,87,88,89,90,91,92,93,94,95,96,97,98,99,100,101,102,103,104,105,106,107,108,109,110,111,112,113,114,115,116,117,118,119,120]).

**Table 3:** Molecular mechanisms and anti-inflammatory targets of botanical extracts in various in vitro models of asthma (2016–2025).

Plant Source	Parts of the Plant	Material Type	Marker	Stimulator	Inhibition Effect	Ref
RAW264.7 cells (Murine macrophage cell line)						
*Salvia plebeian*	Aerial and root	Ethanol extracts	NR	LPS	NO, TNF-α, IL-6	[54]
*Peucedanum japonicum*	Root	Ethanol extract, crude	peujaponiside; pteryxin; hyuganin C; peucedanol derivatives	LPS	NO, PGE2, iNOS, COX-2 TNF-α, IL-6	[55]
*Anthriscus sylvestris*	Root	Root extract, crude	NR	LPS	NO, IL-6	[82]
*Hyssopus cuspidatus*	Aerial	Ethanol extract, crude	rosmarinic acid; hyperoside; salvigenin; diosmin; 3,4-dimethoxycinnamic acid	LPS	NO, TNF-α, IL-6, ROS, p-MAPK, p-NF-κB	[56]
*Physalis peruviana*	Leaf	Methanol extract, crude	NR	LPS	MCP-1	[57]
*Pistacia weinmannifolia*	Root	Root extract, crude	NR	LPS	MCP-1, p-NF-κB	[63]
*Eriobotrya japonica*	Leaf	Leaf extract, crude	NR	LPS	iNOS, COX-2	[58]
*Myxopyrum serratulum*	Leaf	Methanol extract, crude	p-coumaric acid; catechin; naringenin	LPS	NO, ROS, IL-1α, IL-1β, IL-2, IL-6, IL-12, IL-17A, GM-CSF, TNF-α, IFN-γ	[50]
*Angelica reflexa*	Root	Ethanol extract, crude	NR	LPS	NO, iNOS, IL-6	[72]
*Callicarpa japonica*	Aerial	Methanol extract, crude	forsythoside B; verbascoside; samioside	LPS	TNF-α	[48]
*Adenophora Stricta*	Root	Aqueous extract, crude	NR	LPS	IL-1β, IL-6, TNF-α, MCP-1, iNOS, p-JNK, p-NF-κB,	[86]
THP-1 cells (Human monocytic cell line)						
*Shuteria involucrate*	Roots	Ethanol extract, crude	NR	LPS	IL-1β, IL-6, IL-18, MCP-1, TLR4, p-NF-κB	[62]
A549 cells (Human alveolar epithelial cell line)						
*Callicarpa japonica*	Aerial	Methanol extract, crude	forsythoside B; verbascoside; samioside	PMA	IL-6, IL-8, MCP-1, p-NF-κB, p-IκBα	[48]
H292 cells (Human pulmonary mucoepidermoid carcinoma cell line)						
*Lindera obtusiloba*	Leaf	Methanol extract, crude	quercetin rhamnosides; kaempferol rhamnosides	TNF-α	IL-4, IL-5, IL-6, IL-13, p-NF-κB	[83]
*Alnus hirsuta*	Branches	Methanol extract, crude	oregonin	TNF-α	TNF-α, IL-4, IL-5, IL-6, MUC5AC	[52]
*Dictamnus dasycarpus*	Root bark	Aqueous extract, crude	rutaevin; dictamnine; limonin; obacunone; fraxinellone	IL-4/IL-13	MUC5AC, p-STAT3, p-STAT6	[66]
*Inula japonica*	Flower	Aqueous extract + isolated compounds	britannilactone; 6-methoxyluteolin; 1-O-acetylbritannilactone	IL-4/IL-13	p-JNK2, p-STAT3, p-STAT6	[68]
BEAS-2B cells (Human bronchial epithelial cell line)						
*Salvia plebeia*	Aerial and root	Ethanol extracts, crude	NR	LPS/TNF-α	IL-6, IL-8	[54]
*Gynostemma pentaphyllum* [Gypenoside A]		Isolated compound	gypenoside A	IL-4/TNF-α	IL-6, IL-8, MCP-1, CCL5, CCL11, CCL24, ROS	[64]
*Fritillaria unibracteata*	Bulbus	Total alkaloid fraction	peiminine; peimine; edpetiline; khasianine; peimisine; sipeimine	TNF-α, IL-4	TRPV1, NEAT, p-p38	[65]
HMC-1 (Human mast cell line)						
*Dryopteris crassirhizoma*	Rhizome	Ethanol extract, crude	isoquercetin; chlorogenic acid; pinellic acid	PMA/A23187	IL-6 and TNF-α	[70]
*Sophora japonica* [sophoricoside]	Seed	Isolated compound	sophoricoside	anti-DNP-IgE	PGD2, LTB4, LTC4	[71]

Source: Compiled by authors based on the cited literature. Ref, Reference; NR: Not Reported.

**Table 4:** Therapeutic efficacy and systemic immunomodulatory effects of botanical extracts in in vivo asthma models (2016–2025): Part A.

Plant Source	Parts of the Plant	Material Type	Marker	Stimulator	Inhibition Effect	Ref
BALB/c mouse						
*Pistacia integerrima*	Gall	Ethanol extract, crude	NR	OVA	IL-4, IL-5, TNF-α	[75]
*Erythronium japonicum*		Ethanol extract, crude	chlorogenic acid; caffeic acid	OVA		[76]
*Salvia plebeia*	Aerial and root	Ethanol extracts, crude	NR	OVA	IL-4, IL-5, IL-13, mucus	[54]
*Rosae multiflorae Fructus*		Hot water extract, crude	NR	OVA		[69]
*Eclipta prostrata*		Standardized methanol extract	wedelolactone; demethylwedelolactone; oroboside	OVA	IL-13	[77]
*Cnidium monnieri* [Oosthole]		Isolated compound	osthole	OVA	IL-4, IL-5, IL-13, IgE, p-NF-κB	[78]
*Bupleurum chinense*	Root	Root extract, crude	NR	OVA	IL-4, IL-5, IL-1β, IL-6, TNF-α, RORγt, IL-17A, GATA3, IgE, IgG1, IgG2a, p-NF-κB, p-IκBα	[79]
*Artemisia argyi*		Methanol extract + isolated compound	dehydromatricarin A	OVA	IL-4, IL-5, IL-13, IgE, MMP-9, p-ERK	[81]
*Physalis peruviana*	Leaf	Methanol extract, crude	NR	OVA	IL-4, IL-5, IL-13, MCP-1, KEN-5, p-p38, p-JNK, p-NF-κB	[57]
*Pistacia weinmannifolia*	Root	Root extract, crude	NR	OVA	IL-4, IL-5, IL-13, IgE, MCP-1	[63]
*Eriobotrya japonica*	Leaf	Leaf extract, crude	NR	OVA	IL-4, IL-13, IgE, NO, EPO	[58]
*Dryopteris crassirhizoma*	Rhizome	Ethanol extract, crude	isoquercetin; chlorogenic acid; pinellic acid	OVA	IL-4, IL-5, IL-6, IL-13, IgE, IgG1, p-NF-κB	[70]
*Lindera obtusiloba*	Leaf	Methanol extract, crude	quercetin rhamnosides; kaempferol rhamnosides	OVA	IL-4, IL-5, IL-3, MUC5AC, eotaxin, IgE, ROS, NO, TBARS, p-NF-κB, p-NF-κB, p-AP1	[83]
*Myxopyrum serratulum*	Leaf	Methanol extract, crude	p-coumaric acid; catechin; naringenin	OVA	EPO, PGE2, NO, ROS, IL-4, IL-5, IL-13, iNOS, COX-2	[50]
*Scrophularia koraiensis*		Ethanol extract, crude	aucubin; harpagide	OVA	IL-5, IL-13, IgE, iNOS, p-NF-κB	[59]
*Sophora japonica* [sophoricoside]	Seed	Isolated compound	sophoricoside	OVA	IL-4, IL-5, IL-13, IL-17, IFN-γ, TNF-α, IgE, IgG1, IgG2a, histamine, LTC4.	[71]
*Angelica reflexa*	Root	Ethanol extract, crude	NR	OVA	IL-5, IL-13, IgE, eotaxin-3, iNOS IFF4	[72]
*Callicarpa japonica*	Aerial	Methanol extract, crude	forsythoside B; verbascoside; samioside	OVA	TNF-α, IL-6, IgE, histamine, iNOS, p-CREB, p-NF-κB, p-IκBα	[48]
*Alnus hirsuta*	Branches	Methanol extract, crude	oregonin	OVA	IL-4, IL-5, IL-6, IL-13, MUC5AC, eotaxin, IgE, p-MAPK, p-NF-κB, p-IκBα	[52]
*Castanea crenata*	Inner shell	Ethanol extract, crude	ellagic acid	OVA	IL-4, IL-5, IL-13, IgE, MMP-9, iNOS, COX-2, p-NF-κB	[84]
*Phlomis umbrosa*		Ethanol extract, crude	umbroside; shanzhiside methyl ester; seamoside	OVA	IL-4, IL-5, IL-13, IgE, p-ERK, p-NF-κB	[85]
*Artemisia gmelinii*		Extract, crude	NR	OVA	IL-4, IL-5, IL-13, IgE, histamine, GATA-3	[73]

Source: Compiled by authors based on the cited literature. Ref, Reference; NR: Not Reported.

**Table 5:** Therapeutic efficacy and systemic immunomodulatory effects of botanical extracts in in vivo asthma models (2016–2025): Part B.

Plant Source	Parts of the Plant	Material Type	Marker	Stimulator	Inhibition Effect	Ref
BALB/c mouse						
*Gynostemma pentaphyllum* [Gypenoside A]		Isolated compound	gypenoside A	OVA	IL-4, IL-5, IL-6, IL-13, TNF-α, CCL11, CCL24, IgE, IgG1, MDA, COX-2	[64]
*Scrophularia takesimensis*	Root	Ethanol extract, crude	NR	OVA	IL-4, IgE	[74]
*Dictamnus dasycarpus*	Root bark	Aqueous extract, crude	rutaevin; dictamnine; limonin; obacunone; fraxinellone	OVA	IL-4, IL-5, IL-13, IgE, TARC, MDC, IP-10, MUC5AC, FOXA2, p-STAT3, p-STAT6	[66]
*Camellia sinensis*		Ethanol extract, crude	caffeine; epigallocatechin; epicatechin; EGCG; ECG	OVA	IL-4, IL-5, IL-13, IgE, MMP-9, p-NF-κB, p-IκB	[87]
*Alstonia scholaris*	Leaf	Total alkaloid fraction	scholaricine; 19-epischolaricine; vallesamine; picrinine	OVA	IL-4, IL-5, Il-6, IL-8, IL-13, IL-19, IL-17A, IL-25, IL-33, MCP-1, IgE, eotaxin, MUC5AC, ST2, LTB4, LTC4, LTD4, LTE4	[61]
*Melia azedarach*	Fruit	Ethanol extract, crude	toosendanin	OVA	IL-4, IL-5, IL-13, IgE, 8-OHdG, MMP-9	[67]
*Hyssopus cuspidatus*		Chemically characterized crude extract	terpenoids; flavonoids; phenolic acids	OVA	IL-4, IL-5, IL-13, IgE, p-PI3K, p-JNK, p-p38	[88]
*Shuteria involucrate*	Root	Ethanol extract, crude	NR	OVA	IL-4, IL-5, IL-6, IL-9, IL-13, MCP-1, MUC5AC, TLR4, p-NF-κB	[62]
*Inula japonica*	Flower	Aqueous extract + isolated compounds	britannilactone; 6-methoxyluteolin; 1-O-acetylbritannilactone	OVA	IL-4, IL-5, IL-13, IgE, MDC, eotaxin, periostin	[68]
*Phellodendron amurense*	Trunk bark	Methanol extract, crude	phellodendrine; jatrorrhizine; palmatine; berberine; limonin	OVA	IL-4, IL-5, IL-13, TNF-α, CCR3, TARC, IgE,	[89]
C57BL/6 mouse						
*Peucedanum japonicum*	Root	Ethanol extract, crude	peujaponiside; pteryxin; hyuganin C; peucedanol derivatives	OVA	IL-4, IL-5, IL-13, GATA3	[55]
*Anthriscus sylvestris*	Root	Root extract, crude	NR	OVA	IL-4, IL-5, IL-13, IgE, eotaxin-3, iNOS, IRF4	[82]
*Adenophora Stricta*	Root	Aqueous extract, crude	NR	OVA	IL-4, IL-5, IgE	[86]
*Fritillaria unibracteata*	Bulbus	Total alkaloid fraction	peiminine; peimine; edpetiline; khasianine; peimisine; sipeimine	OVA	IL-1β, IL-4, IL-17A, IL-33, TNF-α,,IgE, TRPV1, NFAT, TSLP, p-p38	[65]
Sprague–Dawley (SD) rat						
*Artemisia pallens*	Aerial	Methanol extract, crude	NR	OVA	IL-4, IL-1β, IL-6, TNF-α, TGF-β	[80]
*Hyssopus cuspidatus*	Aerial	Ethanol extract, crude	rosmarinic acid; hyperoside; salvigenin; diosmin; 3,4-dimethoxycinnamic acid	OVA	IL-4, IL-6, IL-17, TNF-α, IgE, eotaxin.	[56]
*Spenceria ramalana*	Whole	50% ethanol polyphenolic extract/fraction	Polyphenolic component	OVA	IL-4, IL-5, IL-13, TNF-α MMP-9, α-SMA	[60]

Source: Compiled by authors based on the cited literature. Ref, reference; NR: Not Reported.

**Table 6:** Major phytochemical classes, identified marker compounds, and associated anti-asthmatic targets in the reviewed botanical extracts.

Phytochemical Class	Representative Metabolites Identified in Reviewed Studies	Representative Source /Extract	Evidence Type	Associated Anti-Asthmatic Targets
Phenolic acids	Chlorogenic acid, caffeic acid, p-coumaric acid, 3,4-dimethoxycinnamic acid	*Erythronium japonicum*, *Dryopteris crassirhizoma*, *Myxopyrum serratulum*, *Hyssopus cuspidatus*	Extract markers	NF-κB, MAPK, Th2 cytokines, ROS, mucus hypersecretion
Flavonoids/flavonoid glycosides	Isoquercetin, hyperoside, quercetin rhamnosides, kaempferol rhamnosides, naringenin, diosmin, salvigenin, 6-methoxyluteolin	*Dryopteris crassirhizoma*, *Lindera obtusiloba*, *Hyssopus cuspidatus*, *Inula japonica*	Extract markers/active compounds	NF-κB, MAPK/AP-1, JAK/STAT3/6, HO-1/NQO1
Catechins/flavanols	Catechin, epigallocatechin, epicatechin, epigallocatechin gallate, epicatechin gallate	*Myxopyrum serratulum*, *Camellia sinensis*	Extract markers	NF-κB/IκBα, MMP-9, IgE, IL-4/IL-5/IL-13
Coumarins/coumestans	Osthole, wedelolactone, demethylwedelolactone, pteryxin, peucedanol derivatives	*Cnidium monnieri*, *Eclipta prostrata*, *Peucedanum japonicum*	Isolated compound/extract markers	NF-κB, IκBα, GATA3, Th2 cytokines, eosinophilic infiltration
Iridoid glycosides	Aucubin, harpagide, shanzhiside methyl ester, seamoside, umbroside	*Scrophularia koraiensis*, *Phlomis umbrosa*	Extract markers	NF-κB, ERK, iNOS, HO-1, Th2 cytokines
Phenylethanoid glycosides	Forsythoside B, verbascoside, samioside	*Callicarpa japonica*	Extract markers	CREB, NF-κB, HO-1, TNF-α, IL-6
Diarylheptanoids	Oregonin	*Alnus hirsute*	Extract marker	MAPK, NF-κB, MUC5AC, Th2 cytokines
Saponins	Gypenoside A	*Gynostemma pentaphyllum*	Isolated compound	Th2 cytokines, chemokines, ROS, IgE/IgG1, COX-2
Steroidal/indole/isoquinoline alkaloids	Peiminine, peimine, edpetiline, khasianine, peimisine, sipeimine; scholaricine, vallesamine, picrinine; berberine, palmatine, jatrorrhizine	*Fritillaria unibracteata*, *Alstonia scholaris*, *Phellodendron amurense*	Total alkaloid fraction/extract markers	TRPV1/Ca^2+^/NFAT, TSLP, p38, Th2/Th17 cytokines, IgE
Limonoids/triterpenoids	Limonin, obacunone, fraxinellone, rutaevin, toosendanin	*Dictamnus dasycarpus*, *Melia azedarach*, *Phellodendron amurense*	Extract markers	STAT3/6, FOXA2, MUC5AC, oxidative stress, MMP-9
Sesquiterpene lactones	Britannilactone, 1-O-acetylbritannilactone, dehydromatricarin A	*Inula japonica*, *Artemisia argyi*	Isolated compound/extract marker	JAK2/STAT3/6, ERK, MMP-9, Th2 cytokines

Abbreviations: ROS, reactive oxygen species; MUC5AC, mucin 5AC; TSLP, thymic stromal lymphopoietin. “Extract marker” indicates that the compound was identified in the active extract but was not necessarily tested individually in the asthma model. “Isolated compound” indicates that the compound itself was evaluated pharmacologically.

**Table 7:** Quantitative stratification of tested materials and chemical characterization across the 39 reviewed studies.

Tested Material Category	Definition Used in This Review	Representative Examples	Number of Studies	Proportion
Crude extracts without reported phytochemical markers	Crude botanical extracts tested without specific marker compounds, chromatographic profiling, or quantitative phytochemical analysis	*Pistacia integerrima*, *Salvia plebeia*, *Rosae multiflorae Fructus*, *Bupleurum chinense*, *Artemisia pallens*, *Anthriscus sylvestris*, *Physalis peruviana*, *Pistacia weinmannifolia*, *Eriobotrya japonica*, *Angelica reflexa*, *Artemisia gmelinii*, *Adenophora stricta*, *Scrophularia takesimensis*, *Spenceria ramalana*, *Shuteria involucrata*	15	38.50%
Chemically characterized crude extracts	Crude extracts accompanied by phytochemical marker identification, HPLC/LC-MS profiling, or broad phytochemical class characterization, but without full standardization criteria	*Erythronium japonicum*, *Eclipta prostrata*, *Peucedanum japonicum*, *Hyssopus cuspidatus*, *Dryopteris crassirhizoma*, *Lindera obtusiloba*, *Myxopyrum serratulum*, *Scrophularia koraiensis*, *Callicarpa japonica*, *Alnus hirsuta*, *Castanea crenata*, *Phlomis umbrosa*, *Dictamnus dasycarpus*, *Camellia sinensis*, *Melia azedarach*, *Hyssopus cuspidatus*, *Phellodendron amurense*	17	43.60%
Standardized extracts	Extracts with defined quantitative marker specifications, batch-to-batch reproducibility, or explicit standardization criteria	Not clearly reported in the reviewed studies, unless confirmed by re-checking the original articles	0 or not clearly identifiable	0% or not applicable
Extract plus isolated constituent(s)	Studies evaluating both a botanical extract and isolated compounds derived from or associated with that extract	*Artemisia argyi extract* plus dehydromatricarin A; *Inula japonica extract* plus britannilactone, 6-methoxyluteolin, and 1-O-acetylbritannilactone	2	5.10%
Enriched fractions	Partially purified preparations enriched in a specific phytochemical class	*Fritillaria unibracteata total alkaloids*; *Alstonia scholaris total alkaloids*	2	5.10%
Isolated compounds only	Purified single plant-derived constituents tested as the main intervention	Osthole; sophoricoside; gypenoside A	3	7.70%
Total			39	100%

Note: Chemically characterized crude extracts were not classified as standardized extracts unless quantitative marker criteria, batch reproducibility, or chemical specifications were reported. This table focuses on the chemical definition of tested materials; extraction solvent, yield, polarity/fraction type, and extraction method are summarized separately in Table 8.

**Table 8:** Extraction-related variables reported across the reviewed anti-asthmatic botanical studies.

Variable	Recommended Extraction from Original Studies	Reporting Status in This Review	Relevance
Plant part used	Leaf, aerial part, root, rhizome, flower, fruit, seed, bark, gall, bulb, whole plant	Reported for most studies	Determines phytochemical composition and biological interpretation
Extraction solvent	Water, ethanol, methanol, hydroethanol, ethyl acetate, *n*-butanol, chloroform, petroleum ether, etc.	Summarized in Table 2, Table 3 and Table 4 where available	Determines enrichment of hydrophilic vs. lipophilic constituents
Extraction yield	% yield or mg extract/g dried material	Often not reported; record as NR when unavailable	Required for reproducibility and dose translation
Preparation type	Crude extract, chemically characterized crude extract, standardized extract, enriched fraction, isolated compound	Already partly summarized in Table 6	Indicates degree of chemical definition
Polarity/fraction nature	Hydrophilic, hydroalcoholic/intermediate, lipophilic, enriched fraction, isolated compound	Newly summarized in this review	Helps interpret whether phenolics/glycosides or lipophilic terpenoids/alkaloids may dominate
Phytochemical characterization	None, marker identified, HPLC/LC-MS profile, quantified marker, standardized specification	Partly summarized in Table 5 and Table 6; linked to extraction solvent where available	Supports chemical reproducibility and standardization
Fractionation procedure	Liquid–liquid partition, column fraction, total alkaloid fraction, purified compound	Report if available	Distinguishes crude extract from partially purified fraction

Note: NR indicates that the information was not reported in the original study. Extraction polarity was inferred from the reported solvent or preparation type and should be interpreted cautiously when solvent ratios, fractionation procedures, or yield data were unavailable.

## Data Availability

The original contributions presented in this study are included in the article. Further inquiries can be directed to the corresponding author.

## Abbreviations

The following abbreviations are used in this manuscript:

AECs	Airway epithelial cells
Th2	Type 2 helper T cells
TLRs	Toll-like receptors
NF-κB	Nuclear factor kappa B
AP-1	Activator protein-1
IL	Interleukin
TSLP	Thymic stromal lymphopoietin
EMT	epithelial–mesenchymal transition
TGF-β	Transforming growth factor-β
IgE	Immunoglobulin E
AHR	Airway hyperresponsiveness
B cells	B lymphocytes
FcεRI	High-affinity receptors
PGD2	Prostaglandin D2
LPS	Lipopolysaccharide
PMA	Phorbol 12-myristate 13-acetate
ICAM-1	Intercellular adhesion molecule-1
NO	Nitric oxide
EE	Ethanol extract
FE	Fruit extract
ME	Methanol extract
RE	Root extract
IRF4	Interferon regulatory factor 4
ROS	Reactive oxygen species
MCP-1	Monocyte chemoattractant protein-1
MMP	Matrix metalloproteinase
LE	Leaf extract
EPO	Eosinophil peroxidase
AE	Aqueous extract
TA	Total alkaloids
ST2	Suppression of tumorigenicity 2
SOD	Superoxide dismutase
GSH	Glutathione
MDA	Malondialdehyde
Penh	Enhanced pause
